# Induced aneuploidy in neural stem cells triggers a delayed stress response and impairs adult life span in flies

**DOI:** 10.1371/journal.pbio.3000016

**Published:** 2019-02-22

**Authors:** Mihailo Mirkovic, Leonardo G. Guilgur, Alexandra Tavares, Diogo Passagem-Santos, Raquel A. Oliveira

**Affiliations:** Instituto Gulbenkian de Ciência, Oeiras, Portugal; Stony Brook University, UNITED STATES

## Abstract

Studying aneuploidy during organism development has strong limitations because chronic mitotic perturbations used to generate aneuploidy usually result in lethality. We developed a genetic tool to induce aneuploidy in an acute and time-controlled manner during *Drosophila* development. This is achieved by reversible depletion of cohesin, a key molecule controlling mitotic fidelity. Larvae challenged with aneuploidy hatch into adults with severe motor defects shortening their life span. Neural stem cells, despite being aneuploid, display a delayed stress response and continue proliferating, resulting in the rapid appearance of chromosomal instability, a complex array of karyotypes, and cellular abnormalities. Notably, when other brain-cell lineages are forced to self-renew, aneuploidy-associated stress response is significantly delayed. Protecting only the developing brain from induced aneuploidy is sufficient to rescue motor defects and adult life span, suggesting that neural tissue is the most ill-equipped to deal with developmental aneuploidy.

## Abstract 2

A novel genetic tool for inducing chromosomal imbalance in fruit flies in an acute and time-controlled manner reveals that the brain is the most sensitive tissue when it comes to coping with abnormal karyotypes during development.

## Author summary

Aneuploidy—the presence of an abnormal number of chromosomes in a cell—is a hallmark of cancer and developmental disorders. However, it is notoriously difficult to study in a living organism. We have thus developed a new genetic tool that allows for the inducible generation of aneuploidy in the fruit fly *Drosophila melanogaster* at any developmental stage. The tool is based on reversible depletion of the protein cohesin, a major regulator of fidelity of chromosome segregation during cell division. Contrary to our expectations, when larvae are challenged with organism-wide mosaic aneuploidy, they still hatch into adult flies, albeit with severe motor defects and reduced life span. While most of the developing epithelial tissues respond to aneuploidy by inducing cell death, eliminating the cells with an abnormal number of chromosomes, the developing brain does not. Most of the aneuploid neural stem cells can keep proliferating despite their abnormal chromosomal number and chromosomal instability, suggesting that these cells are uniquely resistant to aneuploidy-associated stresses. The differential tissue response led us to hypothesize that the brain is the limiting tissue in response to developmental aneuploidy. To test it, we modified the aneuploidy induction system to protect the brain from aneuploidy while the rest of the tissues were affected. As a result, we observed a complete rescue of previous motor defects and life span reduction, demonstrating that the developing brain is the tissue most susceptible to aneuploidy.

## Introduction

Aneuploidy, a state of chromosome imbalance, was observed over a century ago. Since then, numerous studies have shown that aneuploidy is largely detrimental both at cellular and organism level. In multicellular organisms, chromosome gain or loss results in lethality or developmental defects [1,2]. At the cellular level, studies in yeast and cell culture have demonstrated that aneuploidy has a high fitness cost for the cell because unbalanced karyotypes lead to the activation of multiple stress-response pathways, resulting in reduced proliferation, cell-cycle arrest, or cell death (reviewed in [3]). Cellular stress induced by aneuploidy seems at odds with the hypothesized role of aneuploidy in promoting malignancy, as well as its reported role as a driving force of yeast fitness and evolution [4–6]. Ninety percent of solid tumors harbor whole-chromosome gains and/or losses [7]. Therefore, aneuploidy and its effects on cell fitness and proliferation are context dependent, which emphasizes our need for a better understanding of the immediate and ultimate consequences of this abnormal cellular condition in metazoan tissues and through development.

Study of aneuploidy in vivo is challenging because somatic aneuploidy is a rare event, difficult to capture and trace in real time because of several constraints: i) cells are equipped with surveillance mechanisms that prevent chromosome mis-segregation, making naturally occurring aneuploidy events virtually impossible to evaluate; ii) experimentally induced aneuploidy, by compromising mitotic fidelity, is often of low prevalence, as has been demonstrated for several mammalian [8,9] and *Drosophila* tissues [10–13]; and iii) induction of somatic or constitutional aneuploidy in metazoans relies on chronic mitotic perturbation (listed in [14]), which usually causes embryonic lethality (reviewed in [15]) as a result of progressive accumulation of damage in the developing organism. Thus, from these studies, it is impossible to disentangle short- and long-term consequences of aneuploidy or to examine the kinetics of the aneuploid state response during development. To circumvent these limitations, we generated a genetic system with the power to induce aneuploidy in an acute and time-controlled manner in all the dividing tissues of the developing *Drosophila*. The tool is based on reversible depletion of cohesin, a key molecule regulating mitotic fidelity [16,17]. Cohesin is a tripartite ring complex, composed of Structural Maintenance of Chromosome subunits (SMCs) SMC 1 and SMC3 and the bridging kleisin subunit Double-strand-break repair protein rad21 homolog (RAD21) [18,19]. The primary mitotic role of cohesin is to mediate sister chromatid cohesion by topologically entrapping DNA fibers from neighboring chromatids [20,21]. Cells entering mitosis with premature loss of cohesin and sister chromatid separation activate the Spindle Assembly Checkpoint (SAC), resulting in prolonged mitosis [17,22]. During this SAC-dependent mitotic delay, chromosomes are shuffled from one cell pole to the other by the mitotic spindle [22,23]. Consequently, chromosome shuffling induces genome randomization and aneuploidy upon mitotic exit with a theoretical rate of nearly 100%. Our engineered system enables a quick restoration of cohesin shortly after its inactivation, thereby restricting mitotic abnormalities to a short time frame concomitantly with the generation of high levels of aneuploidy. The acute and controlled nature of the tool allows us to dissect the kinetics of aneuploidy response across various tissue types and developmental stages.

## Results

### A genetic system for acute and time-controlled generation of aneuploidy in a developing organism

**Fig 1:**
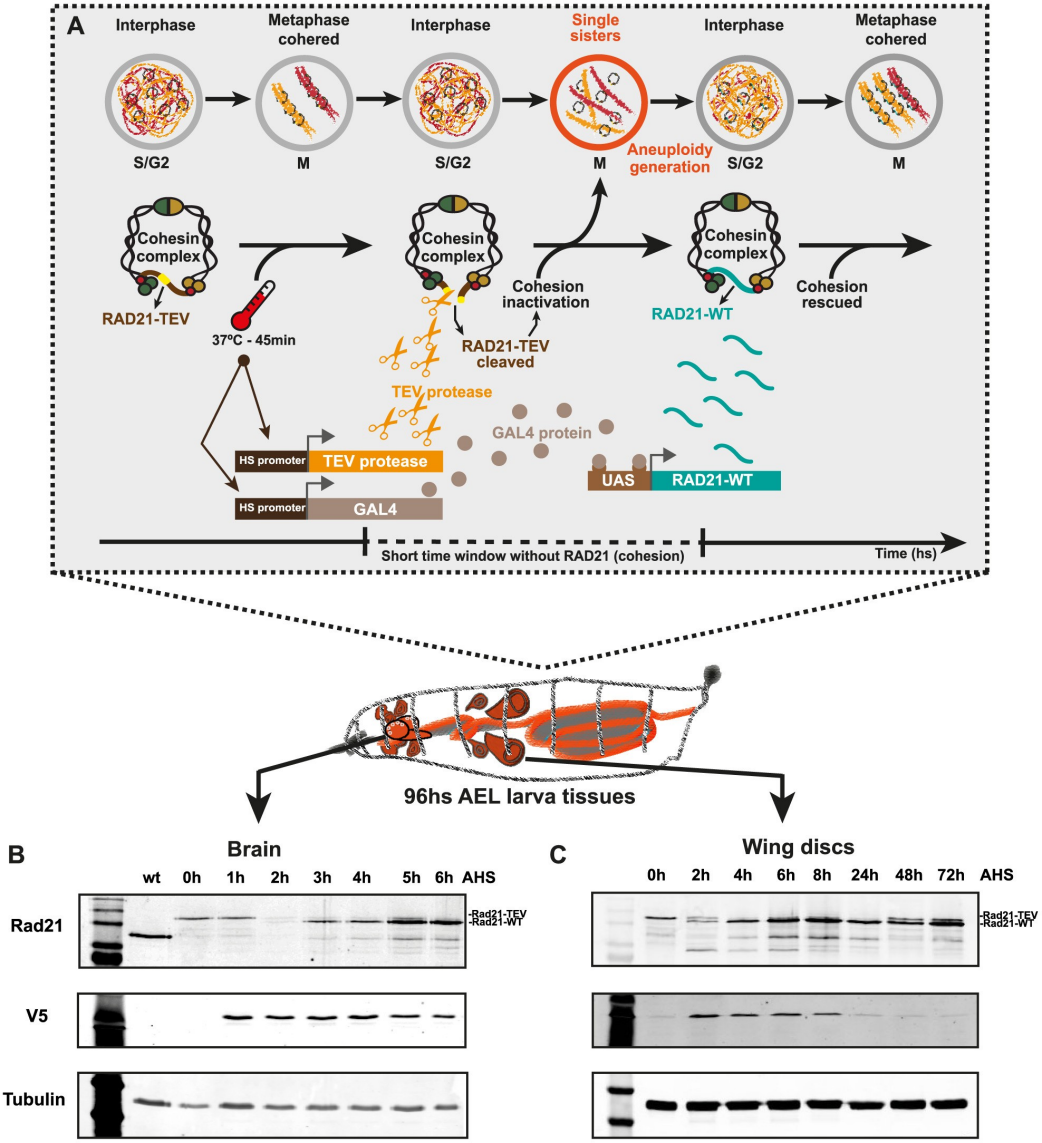
Reversible system for acute cohesin depletion and generation of aneuploidy in the developing *Drosophila*. (A–C) (A) The system relies on the cleavage of a RAD21 cohesin subunit (RAD21-TEV) by the HS-induced expression of TEV protease. Cohesin cleavage is rapid, and RAD21-TEV is completely depleted within hours AHS (see western blots 1B, 1C, RAD21 upper band). Cells entering into mitosis with loss of cohesin experience premature sister chromatid separation, chromosome shuffling, and as a consequence, generation of aneuploidy daughter cells with a theoretical rate of nearly 100%. To restrict mitotic failure to a single cell cycle, shortly after aneuploidy has been generated, cohesion is rescued by inducing the expression of RAD21-WT, which is resistant to TEV protease. The RAD21-WT subunit is reliant on HS Gal4 protein expression, coupled to the expression of the UAS-RAD21-WT and occurring within hours AHS (see western blots 1B, 1C, RAD21 lower band). (B and C) Western blots displaying RAD21-TEV cleavage and RAD21-WT restoring cohesin function in the third-instar larvae brains and wing discs. Tissues samples were taken at different hours AHS. V5 antibody evidences the TEV protease production (from *hspr-nlsV5TEV*), and tubulin antibody was used as loading control. AEL, after egg laying; AHS, after heat-shock induction; GAL4, yeast transcription activator protein Gal4; G2, Gap 2 phase; HS, heat shock; *hspr-nlsV5TEV*, heat shock promoter-nuclear localization signal V5 epitope Tobacco Etch Virus; M, Mitosis; RAD21, Double-strand-break repair protein rad21 homolog; S, Synthesis phase; TEV, Tobacco Etch Virus; UAS, upstream activating sequence; WT, wild type.

To induce aneuploidy in an acute and time-controlled manner, we developed a genetic system based on rapid removal of cohesin complex, the molecular glue that holds sister chromatids together. To prevent chronic cohesin depletion and restrict mitotic failure to a single cell cycle, cohesin depletion is followed by subsequent cohesin rescue. The system relies on the artificial cleavage of a modified version of the RAD21 cohesin subunit that contains Tobacco Etch Virus (TEV) protease cleavage sites (RAD21-TEV) [24]. As previously shown, this system is very efficient at inactivating cohesin upon synthesis of the exogenous TEV protease induced by a heat-shock promoter (HSprom), resulting in long-term inactivation of this complex (>24 hours) [22–24]. To restrict cohesin depletion, we modified this system by promptly rescuing cohesin integrity through the expression of a TEV-resistant RAD21 protein, RAD21-wild type [WT], right after TEV-mediated inactivation. For this purpose, RAD21-WT expression is under the control of upstream activating sequence (UAS) promoter (*UAS-Rad21-wt-myc*) that is induced by the yeast transcription activator protein Gal4 (Gal4) induced concomitantly with the TEV protease (also under a HSprom) (Fig 1A). Given that the TEV protease is under the direct control of HSprom, whereas RAD21-WT relies on a dual expression system (*Gal4-UAS*), we anticipated that the temporal delay in RAD21-WT expression relative to the induction of TEV protease would lead to a short time window of cohesin inactivation (RAD21 cleavage) (Fig 1A).

To test this, we probed for the kinetics of TEV-mediated cleavage of RAD21-TEV and synthesis of RAD21-WT in different tissues of the developing larvae. After heat shock, both *Drosophila* larvae brains and wing discs showed similar kinetics of the TEV-sensitive RAD21 disappearance, followed by the appearance of RAD21-WT (Fig 1B and 1C). The timing of protein depletion/re-establishment differs slightly among different tissues or developmental stages but leads on average to a period of approximately 1 hour without cohesin (Fig 1B, 1C and S1D Fig).

### Reversible removal of cohesin results in a single round of mitotic abnormalities and consequent aneuploidy

The cohesive function of cohesin is established in the Synthesis (S)-phase, concomitantly with DNA replication. Once stabilized on the replicated genome, cohesive cohesin complexes do not turn over [25]. As such, loss of cohesin using our system will affect sister chromatid cohesion in all cells that are in S/Gap 2/Mitosis (S/G2/M) phases during the short period between TEV protease presence and synthesis of RAD21-WT (Fig 1A). In addition to its canonical cohesive function, cohesin has also been implicated in other interphase functions, including regulation of gene expression [26]. In contrast to the cohesive pool, these cohesin molecules are known to be highly dynamic [25,27]. Moreover, recent studies report that cohesin-mediated loops are quickly restored upon cohesin re-establishment [28]. We therefore anticipated that this function should not be severely affected by our system. In sharp contrast, mitotic errors induced upon cohesin cleavage are irreversible because there is no way to restore cellular ploidy after a compromised round of mitosis.

Whereas canonical chronic mitotic perturbations lead to several rounds of mitotic failures, our novel genetic system should lead to cohesion defects only in the first mitosis following the heat-shock because the expression of RAD21-WT should be able to rescue cohesion in the subsequent cell cycle (Fig 1A). To confirm that our genetic system works as anticipated, we focused our analysis on two different cycling tissues from the larva: the developing brain and the epithelial wing discs.

The developing brain of *Drosophila* is an excellent model to study the consequences of developmental aneuploidy. The well-characterized cell lineages, in combination with our tractable system to induce mis-segregation of chromosomes, offer a unique opportunity to trace the fate of aneuploid cells in real time and analyze their effect on the nervous system development. Through larval development, approximately 100 large neural stem cells called Neuroblasts (Nbs) [29] located in the central brain (CB) region divide asymmetrically to self-renew and generate distinct neuronal lineages via differentiating progeny [30].

We evaluated, by live-cell imaging, mitotic fidelity in these Nbs using two independent criteria to estimate the state of sister chromatid cohesion: i) the presence of prematurely separated sister chromatids (single sisters) as opposed to metaphase chromosome alignment and ii) the time cells spend in mitosis, given that premature loss of sister chromatid cohesion is known to activate the SAC and delay mitotic exit [22].

**Fig 2:**
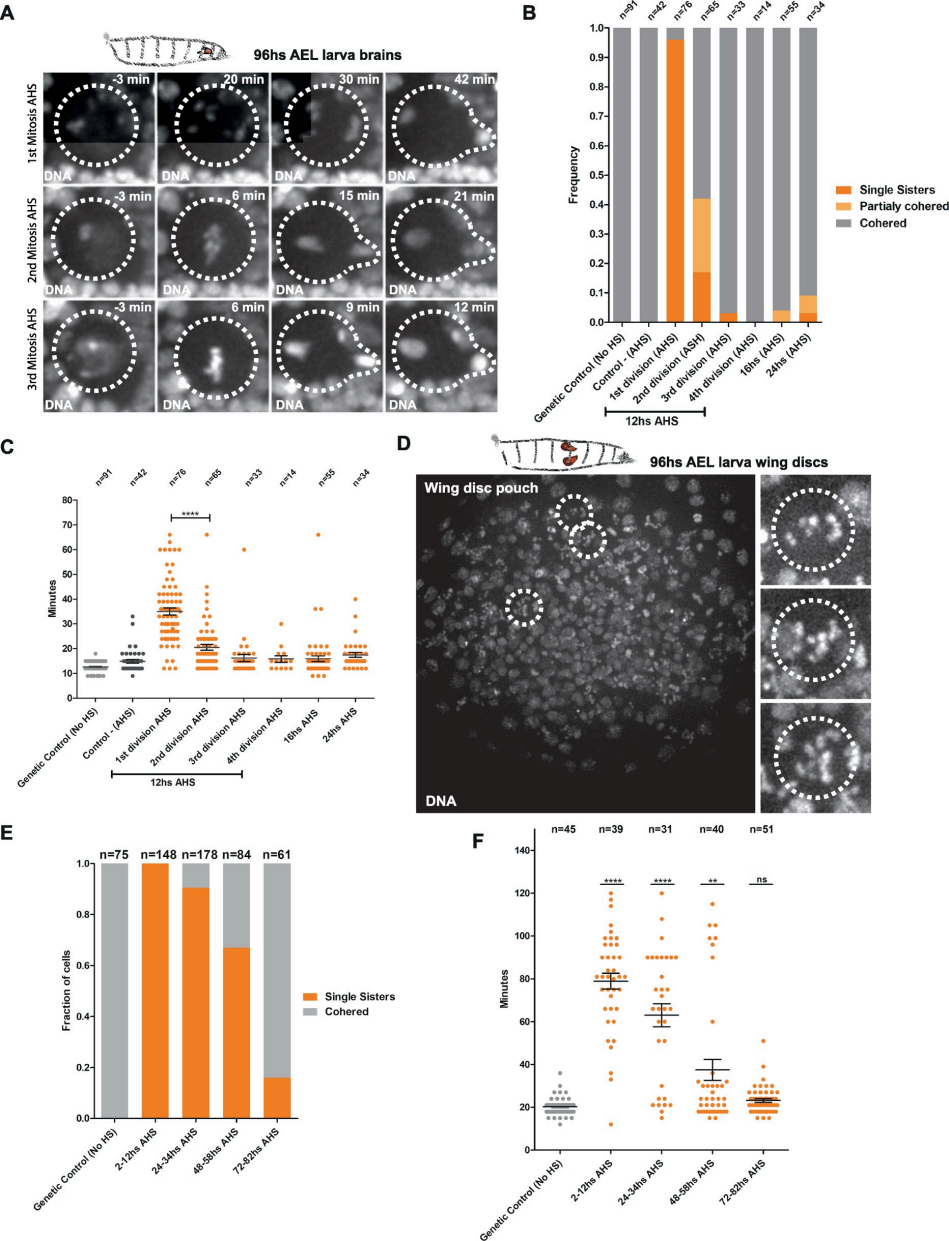
Kinetics of reversible loss of cohesin in the third-instar brains and the wing discs. (A) Stills from live imaging of Nbs AHS (full movies available in supplementary S1 and S2 Movies). Transient loss of cohesion results in a round of defective mitosis and genome shuffling (first division AHS). After this round of division, the following mitosis (second and third divisions AHS) shows the restoration of cohesin function and mitotic fidelity in larvae Nbs. Dashed circles display the mitotic figures of the dividing Nbs. (B) Quantification of cohesive states of third-instar larvae Nbs following RAD21-TEV cleavage and RAD21-WT restoration of cohesin function. More than 80% of the second divisions AHS are already totally or partially cohered. (C) Quantification of mitotic timing and delay caused by SAC activation after RAD21-TEV cleavage and RAD21-WT rescue in the third-instar Nbs (2 to 24 hours AHS). Second divisions AHS evidence a significant reduction in the mitotic timing as a consequence of the rescue of cohesin function. ****P < 0.0001. (D) Cohesin cleavage in the wing disc of third-instar larvae. Stills from live imaging showing single chromatids during mitosis. Dashed circles display epithelial cells from the wing disc pouch undergoing mitosis with loss of cohesion. (E) Quantification of cohesive states of divisions in the third-instar larvae wing disc following RAD21-TEV cleavage and RAD21-WT rescue from 2 hours to 80 hours AHS. Given the long cell cycle and the heterogeneous rate of division of cells in this tissue, the presence of single sisters can still be observed up to 72 hours AHS. (F) Quantification of mitotic timing and delay caused by SAC activation after Rad21-TEV cleavage and Rad21-WT rescue in the third-instar wing discs from 2 to 80 hours AHS. In all panels, *n* = number of cells. ****P < 0.0001; **P < 0.01. Individual numerical values for the presented graphs can be found in S1 Data. AEL, after egg laying; AHS, after heat-shock induction; HS, heat shock; Nb, Neuroblast; RAD21, Double-strand-break repair protein rad21 homolog; SAC, Spindle Assembly Checkpoint; TEV, Tobacco Etch Virus; WT, wild type.

As expected, the first division after the heat shock results in full cohesin cleavage in Nbs, followed by cohesin rescue in subsequent divisions (S1 and S2 Movies). The fast cell cycle of Nbs, coupled with continued proliferation of these cells despite their abnormal genome content (further discussed below), enables analysis of mitotic fidelity throughout several consecutive divisions in great detail. Consistently, in the first mitosis after heat-shock induction (AHS), 95% of Nbs contain single sisters and exhibit mitotic delay and chromosome shuffling (Fig 2A and 2B). However, in the subsequent mitosis, cohesion is restored in approximately 80% (60% fully rescued, 20% intermediate rescued) of the Nbs, with clear metaphases and a shorter mitotic delay (Fig 2A, 2B and 2C). Finally, during the third cell division AHS, the mitotic timing and the cohesive state of Nbs are comparable to heat-shocked controls (Fig 2A, 2B and 2C). Similar results were obtained for larvae heat-shocked at earlier stages of development (S1A, S1B, and S1C Fig).

In contrast to the Nbs, in the epithelial cells of the wing disc, we observe the presence of single sisters even at 48 hours AHS, despite high levels of RAD21-WT protein (Figs 1C, 2D; 2E and 2F). These findings are consistent with the heterogeneity in cell-cycle duration of the wing disc cells, with some cells exhibiting a cell cycle of approximately 48 hours [31,32]. The high frequency of cells in S/G2 phases in this tissue, quantified by the fly Fluorescence Ubiquitination Cell Cycle Indicator (FUCCI) system [33] (S3B Fig), further supports that a high number of cells are affected despite the short time of cohesin inactivation. Note that cohesion establishment is restricted to replication, and consequently, any cell in which RAD21 was depleted and rescued postreplication is unable to “rescue” cohesion despite the presence of the WT RAD21 protein.

To validate the ability of our tool to induce aneuploidy in an acute manner in epithelial tissues, we examined the events following the reversible loss of cohesin. In *Drosophila* epithelial cells, multiple cellular insults, including aneuploidy, can trigger the apoptotic cascade [10,34]. In agreement with these studies, 24 hours AHS in the wing disc, Cleaved Caspase 3 (CC3) staining reveals a large population of dying cells (S2A and S2B Fig). However, at 48 hours AHS, the number of dying cells decreases significantly if cohesin is brought back, but not if the cohesin depletion by TEV is long-term (S2A and S2B Fig). These results suggest that tissue recovery is limited and only possible if the mitotic disruption is restricted in time (or number of cell cycles). Although quantitative analysis was performed exclusively for wing disc epithelial cells and brain Nbs, analysis of other epithelial dividing tissues of the *Drosophila* larvae reveal a similar high incidence of single sisters 3 hours AHS, implying that our system is able to induce a reversible whole-organism loss of cohesin (S3A Fig).

### Larvae challenged with aneuploidy during development hatch into impaired adults

**Fig 3:**
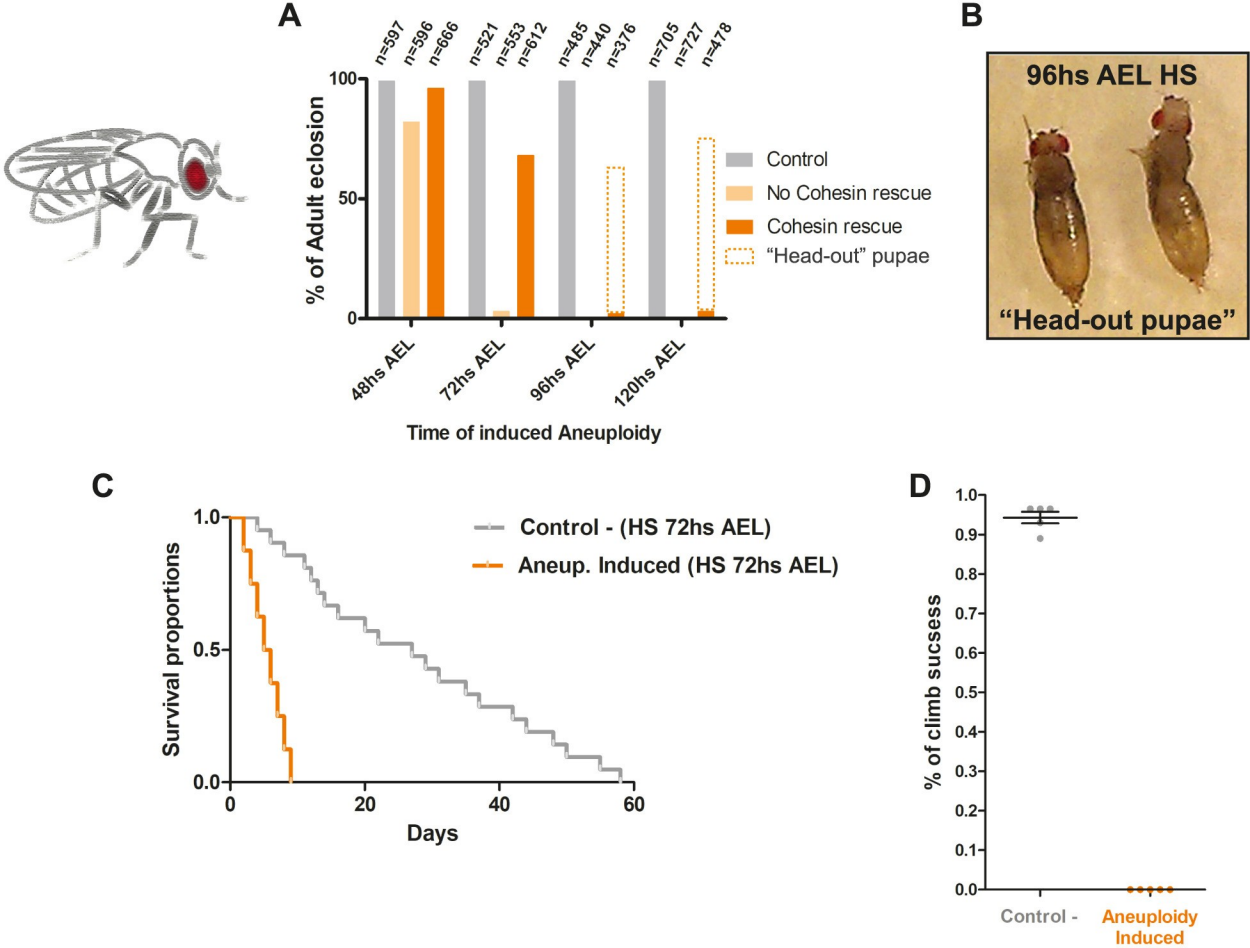
Larvae challenged with organism-wide aneuploidy hatch into adult flies with severe motor defects and reduced life span. (A) Percentages of adult eclosions according to the stage of development at which reversible loss of cohesin (aneuploidy) was inducted (48, 72, 96, and 120 hours AEL). *n* = number of flies. (B) Picture showing “head-out pupae” flies as a result of 96-hours-AEL–induced aneuploidy. (C–D) (C) Kaplan–Meier survival curves showing fractional survival as a function of time. Ecloded flies with 72-hours-AEL–induced aneuploidy showed reduced life span when compared with control flies (only heat-shocked). (D) Climbing assay comparing adult flies with 72-hours–induced aneuploidy and control flies. Percentage of climb success was plotted over the halfway point (10 cm). Ecloded flies with 72-hours-AEL–induced aneuploidy showed impaired motor behavior. Individual numerical values for the presented graphs can be found in S1 Data. AEL, after egg laying; HS, heat shock.

To understand how the organism responds to such high degree of aneuploidy, we traced the larvae through development after cohesin cleavage. For comparative analysis, we monitored eclosion rates for organisms with the long-term TEV protease cleavage system (inducing cohesin removal for >24 hours) and our newly developed system with reversible inactivation of cohesin. Both systems represent a strong injury for all the dividing tissues of the larva; therefore, we expected them to be lethal in the pupa-to-adult transition. However, in contrast to several studies using chronic mitotic perturbations [11,35], flies challenged with aneuploidy using our reversible mitotic perturbation ecloded into adult flies at high frequency (Fig 3A and S3 Movie). Eclosion rates of adults were dependent on the developmental stage at which cohesin was reversibly cleaved (Fig 3A). Early induction of aneuploidy at 48 hours after egg laying (AEL) resulted in eclosion both with and without cohesin rescue. However, with 72-hours–AEL heat shock, there was almost no eclosion if the RAD21 protein subunit was not brought back (Fig 3A). If the larvae were heat-shocked 96 hours AEL, no cohesin rescue resulted in dead pupae, while cohesin rescue resulted in flies trying to escape the pupa but unable to do so (“head-out pupae”) (Fig 3A and 3B). These differences in developmental response to aneuploidy are likely due to increase of cell proliferation during larval development [11,36] (S1A–S1C Fig). Regardless of the developmental stage, all flies that ecloded into adults after the aneuploidy challenge were unable to fly or move normally, even when showing serviceable wings and appendages (Fig 3D and S3 and S4 Movies). Consequently, these flies exhibited markedly shorter lifespans than their control counterparts (Fig 3C). Notably, even when aneuploidy is induced at earlier stages (48 hours AEL), thereby affecting fewer Nbs (S1A Fig), adults displayed a “lethargic” behavior (S5 Movie) while having an otherwise healthy adult morphology.

### A few cell cycles are sufficient to induce chromosomal instability in aneuploid Nbs

**Fig 4:**
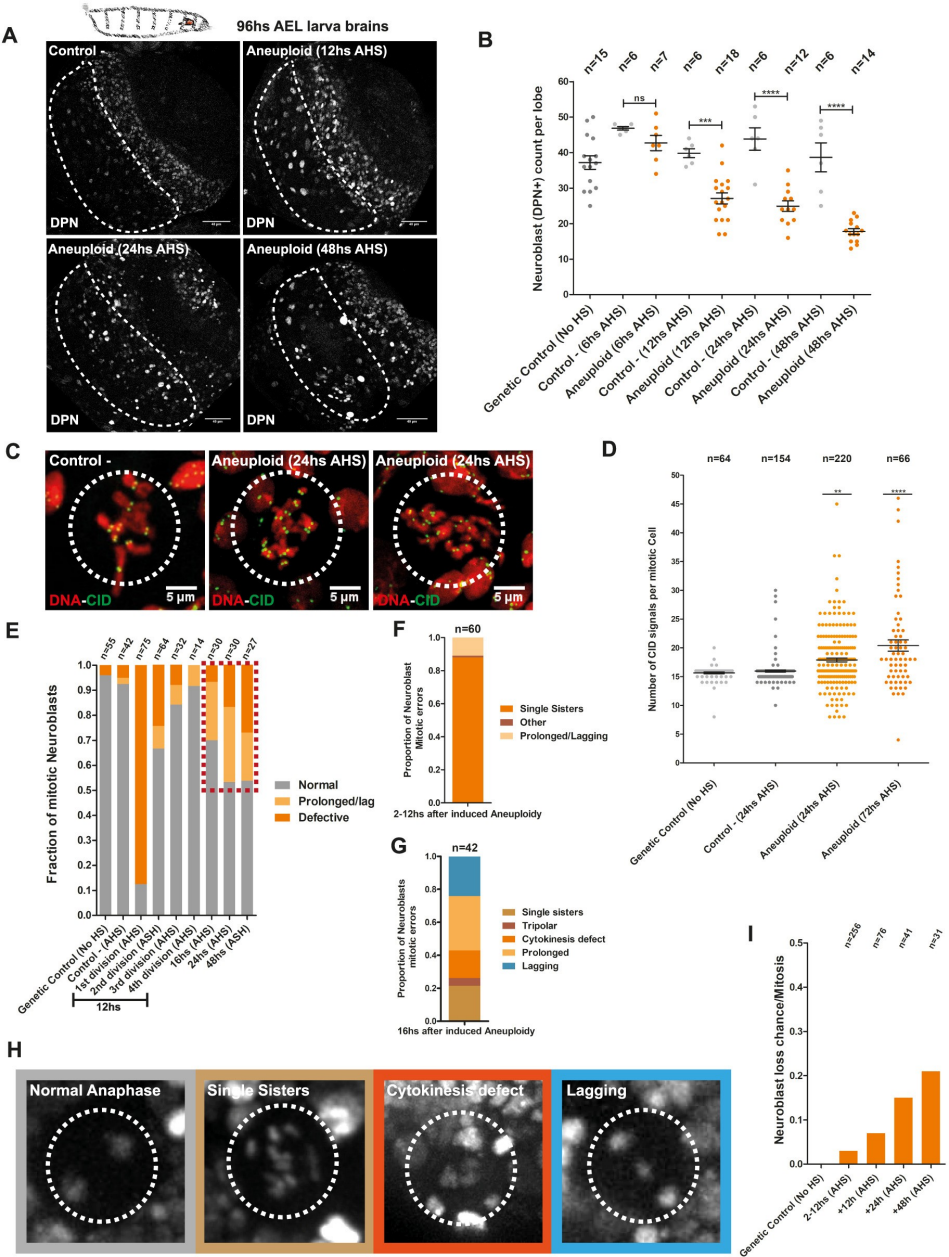
Aneuploidy results in Nb loss, chromosomal instability, and accumulation of chromosomes. (A–B) (A) Nb counts at the CB (dashed shape) in third-instar lobe brains assessed by immunofluorescence with the Nbs marker DPN. (B) Nbs were quantified based on the correlation between morphology and positive signal for DPN at 12, 24, and 48 hours AHS. Reversible loss of cohesin and aneuploidy are followed by a reduction in Nb numbers but not a complete loss of the neural stem-cell pool. *n* = number of lobe brains. ***P < 0.001; ****P < 0.0001. Scale bar = 40 μm. (C–D) (C) Chromosome counts were assessed by CID immunofluorescence (centromere counts) in third-instar Nbs arrested at metaphase with colchicine (dashed circle) at 24 and 72 hours AHS. (D) Aneuploid Nbs accumulate chromosomes through time. *n* = number of cells. ****P < 0.0001; **P < 0.01. Scale bar = 5 μm. (E) Assessment of mitotic defects after aneuploidy induction from 2 to 48 hours AHS. Chromosome instability arises shortly after aneuploidy induction (red dashed box). *n* = number of cells. (F–H) (F and G) Profile of mitotic errors as a consequence of reversible cohesin depletion and consequent aneuploidy from 2 to 12 hours AHS and 16 to 48 hours AHS. **(**H) Stills from live imaging documenting mitotic abnormalities in aneuploid Nbs (dashed circles). *n* = number of cells. (I) Graph displaying the rate of catastrophic mitotic events that result in Nb loss from 2 to 48 hours AHS. Chromosomal instability can result in complete loss of Nb morphology. *n* = number of cells. Individual numerical values for the presented graphs can be found in S1 Data. AEL, after egg laying; AHS, after heat-shock induction; CB, central brain; CID, Histone H3-like centromeric protein; DPN, Deadpan; HS, heat shock; Nb, Neuroblast; ns, not significant.

We hypothesized that the severe motor defects in the newly hatched flies are a direct consequence of aneuploidy in the developing larva brain. Recently, it has been proposed that neural stem cells with unwanted karyotypes are eliminated [11,35], yet Nbs were previously shown to be resistant to large variations in ploidy [37,38]. Furthermore, previous studies also reported fly eclosion despite mitotic perturbations in the Nbs [13,39]. To gain insight on the potential fates of the aneuploid Nbs, first we analyzed a possible change in their number after acute aneuploidy induction, using the Nb marker Deadpan (DPN). We quantified all the nuclei with Nb morphology (Nb-like cells) based on their size and location in the CB area. The analysis indicates that there is a gradual decline of the Nb number after the induction of aneuploidy from 12 hours AHS onwards, but never a complete loss of the aneuploid Nb population (Fig 4A and 4B). The slow kinetics and incomplete elimination of the stem-cell population were quite surprising given the extreme levels of aneuploidy generated upon cohesin disruption (approximately 100%).

Premature differentiation and apoptosis were suggested as the main mechanisms of aneuploid Nb elimination, reported in two recent studies [11,35]. However, after acute aneuploidy induction in the entire Nb population, we found a very low frequency of cells undergoing premature differentiation or cell death (S4A and S4B Fig). As a proxy for premature differentiation events, we quantified Nb-like cells that had either lost the DPN marker or abnormally exhibit the differentiation marker Prospero (Pros) with or without coexpression of DPN (S4A and S4B Fig, arrowheads and dashed circles). Pros is the key factor acting as a switch for the transition from stem cell self-renewal to terminal differentiation [40]; therefore, this marker should not be present in Nbs. (S4A Fig). These findings suggest that premature differentiation, although still taking place, is unlikely to be the only form of Nb elimination. To quantify the levels of apoptosis, we evaluated cells positive for cell death markers like CC3 and Death Caspase-1 (DCP1). We found a significant increase in CC3-positive cells in aneuploid brains (S4C and S4D Fig), indicating that apoptosis may also contribute to the elimination of aneuploid cells, as recently proposed [11]. However, CC3 and DCP1 signals rarely correspond to Nb-like cells (S4C, S4D and S4E Fig, arrowheads and dashed circles), suggesting that apoptosis may not be the major cause for Nb elimination. Thus, loss of stem-cell identity and/or cell death are more likely potential consequences of genome randomization rather than specific mechanisms controlling aneuploidy in the neural stem-cell population. Supporting this idea, inhibition of apoptosis by overexpression of the baculovirus protein P35 does not rescue Nb number per brain lobe 24 hours after induction of aneuploidy (S4F Fig).

To dissect the kinetics of the aneuploidy outcomes, we took advantage of the temporal resolution of our system to examine fate of aneuploid cells in real time. We restricted our analysis to third-instar wandering larvae because at this stage, all Nbs are engaged into active cell divisions [30]. Induction of aneuploidy at this developmental stage affects the entire Nb population, which facilitates cell-fate analysis. We observed a significant number of Nbs proliferating for several days and displaying a tendency for chromosome accumulation over time (Fig 4C and 4D). To analyze the number of chromosomes in each dividing Nb, we performed chromosome spreads and counted the number of centromeres per mitotic figure (each chromosome contains two centromere dots in mitosis). A single round of mitosis upon premature loss of sister chromatid cohesion should result in a maximum of 16 chromatids per Nbs, in the rare cases of complete asymmetric segregation (0.0013%). However, chromatid numbers can reach over 32 chromatids per cell, 24 hours and 72 hours after loss of cohesin, at a much higher frequency (Fig 4D). This analysis suggests that chromosome accumulation does not solely result from the initial loss of cohesin.

To investigate this further, we characterized the mitotic fidelity of aneuploid Nbs. As described above, mitotic divisions that immediately follow the initial loss of cohesin do not display major mitotic errors, and most of the defects observed are cohesin-related (as expected from our experimental setup) (Figs 2B, 4E and 4F).

However, 16 hours AHS, aneuploid cells start changing their behavior, and a variety of mitotic defects appear, becoming more frequent over time (Fig 4E and 4G). Detailed characterization of the mitotic defects arising 16 hours after the induction of aneuploidy revealed that the majority of them (approximately 60%) are mild, consisting of either a prolonged metaphase or a lagging chromosome. Yet, the remaining approximately 40% consisted of cytokinesis defects, tripolar spindles, and sister chromatid cohesion defects, which are serious abnormalities that can drastically alter numerical ploidy (Fig 4G and 4H). As an alternative way to estimate mitotic errors, we analyzed micronuclei formation after reversible loss of cohesin and consequent aneuploidy. Micronuclei were assessed by evaluating DNA signal together with LAMIN immunofluorescence in spreads from brain tissues at 8 hours and 24 hours AHS. Aneuploid cells exhibited a higher percentage of micronuclei but only 24 hours AHS, reinforcing our observations that severe mitotic abnormalities appear several hours (>16 hours) after aneuploidy was induced (S5A and S5B Fig).

These results reveal that a few hours are enough for the stable divisions of aneuploid karyotypes to become unstable in vivo, leading to further randomization of the genome, as previously shown in yeast and tissue culture [41,42]. This chromosomal instability contributes to Nb number decline because catastrophic mitotic errors can result in complete loss of Nb morphology and positioning (Fig 4I). All together, these results indicate that transient cohesin loss and aneuploidy induction in the Nbs seem to induce a large spectrum of phenotypes contributing to the gradual Nb number decrease over time rather than specific mechanisms for elimination of abnormal karyotypes, as previously postulated [11,35].

### Karyotype restrictions in the proliferating aneuploid Nb population

**Fig 5:**
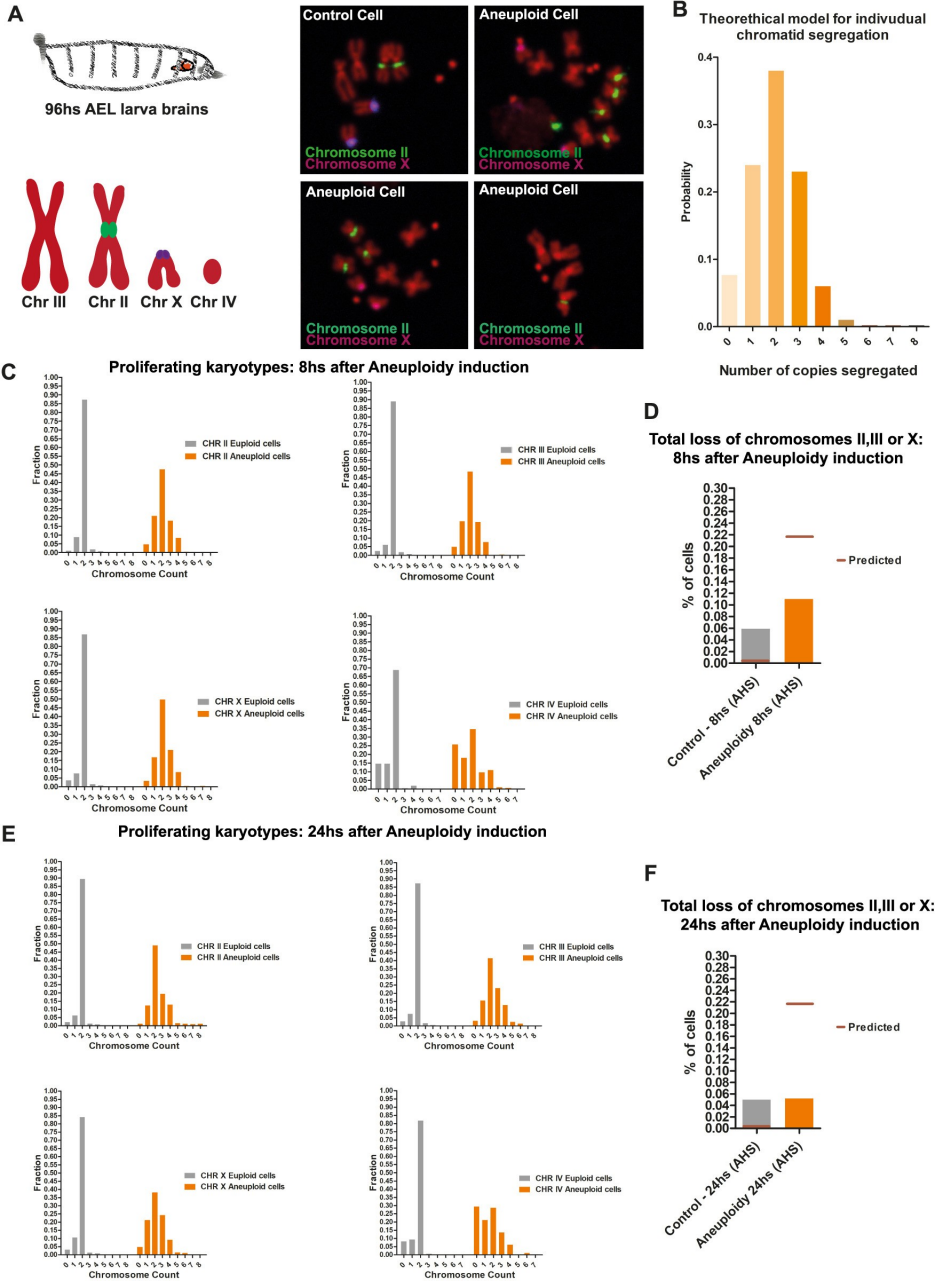
Karyotype restrictions in the proliferating aneuploid Nb population. (A) Panels of FISH of aneuploid Nbs and Control. Schematic of the FISH probe chromosome labeling. (B) Theoretical segregation of sister chromatids of a given chromosome after cohesin loss, assuming segregation to be random (95% in the first mitosis, 20% in the second mitosis). We assumed that each chromatid segregates independently of all other chromatids and with equal probability to the mother and daughter cell. Given this, for one cell division (mitosis) with diploid cells (four chromatids for each chromosome), the distribution of the number of copies of each chromatid per cell is given by a binomial distribution with 4 trials and probability of success of 0.5. (C–D) (C) Frequency distribution of chromosome copy per Nb, 8 hours after aneuploidy induction. (D) Calculated theoretical loss rate for chromosome II, III, or X and the observed frequency of loss of any of these three chromosomes in the proliferating aneuploid Nbs (*n* > 250 over eight brains analyzed per condition). (E–F) (E) Frequency distribution of chromosome copy per Nb, 24 hours after aneuploidy induction. (F) Calculated theoretical loss rate for chromosome II, III, or X and the observed frequency of loss of any of these three chromosomes in the proliferating aneuploid Nbs (*n* > 250 over eight brains analyzed per condition). Individual numerical values for the presented graphs can be found in S1 Data. AEL, after egg laying; AHS, after heat-shock induction; CHR, chromosome; FISH, Fluorescence In Situ Hybridization; Nb, Neuroblast.

To test whether there is a selection of specific karyotypes in the population of dividing aneuploid Nbs, we preformed Fluorescence In Situ Hybridization (FISH) analysis at 8 hours and 24 hours after aneuploidy was induced. To estimate the predicted frequency of specific chromosomes upon cohesin cleavage, we first modeled this process, assuming full random chromosome segregation in a single round, followed by a second round of random segregation in approximately 20% of the cases (based on our experimental observations, see Fig 2B). FISH profiles were then compared with the statistical predictions (Fig 5A and 5B). The FISH profiles confirmed the propensity for chromosome accumulation over time (Fig 5C and 5E). Additionally, this analysis revealed that the karyotypes that can be tolerated by dividing Nbs are restricted to those containing at least one of the major three chromosomes, II, III, or X. The rate of complete loss of these chromosomes in the aneuploidy Nbs population was comparable to the control and thus likely a consequence of experimental error of the FISH (Fig 5D and 5F). We concluded that, although dividing aneuploid Nbs can persist in the tissue, their karyotypes have restrictions, as complete loss of any of the major three chromosomes prevents their proliferation in the developing brain. In contrast, other aneuploid chromosome combinations are compatible with continued proliferation, particularly when chromosomes are gained.

### Reversible loss of cohesin and consequent aneuploidy elicits a stress response in the brain tissue

Our findings reveal that aneuploid cells are not promptly eliminated but instead continue to proliferate within certain karyotype restrictions. This should not only lead to the maintenance of aneuploid stem cells (because of Nb self-renewal) but also to the accumulation of differentiated aneuploid progeny (note that each Nb divides approximately every 2 hours [36]).Therefore, we examined how such increase in aneuploidy within the tissue could affect cellular physiology and influence tissue development.

Several aneuploidy-associated stresses that include oxidative, metabolic, and proteotoxic stress are likely to alter cellular homeostasis [3], leading to p53 activation, cell-cycle arrest/senescence, and in some cases, programed cell death [3,43,44].

**Fig 6:**
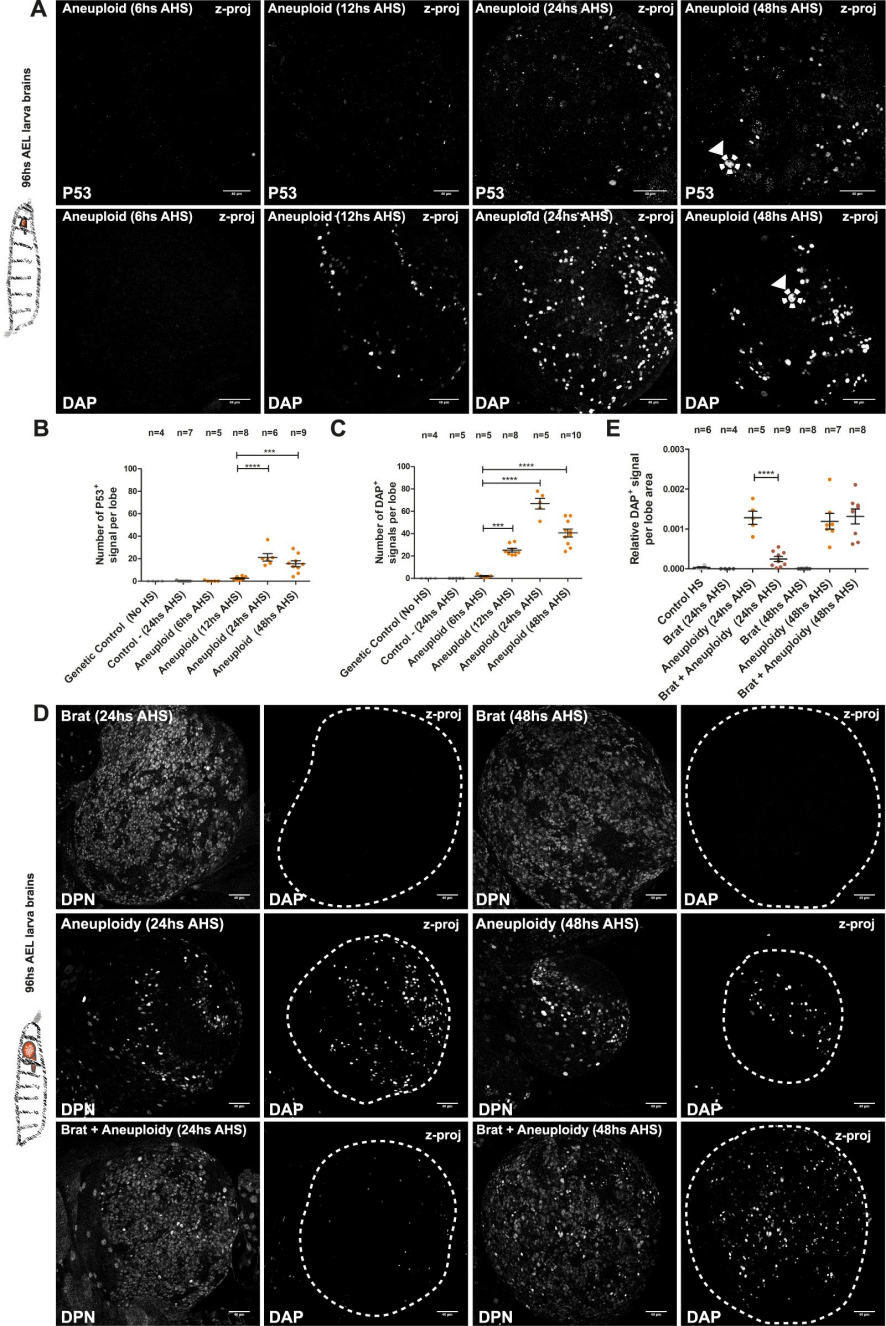
Aneuploidy-induced stress response is delayed in the neural tissue. (A–C) (A) Kinetics of the aneuploidy-induced stress response at 6, 12, 24, and 48 hours AHS; assessed by immunofluorescence of canonical markers P53 and DAP. Nbs show a delayed aneuploidy stress response at 48 hours AHS (arrowheads with dashed circles). (B and C) Counts of overall numbers of p53 and DAP signals per lobe, displaying a significant increase from 24 hours AHS. ***P < 0.001; ****P < 0.0001. Scale bar = 40 μm. (D–E) (D) Pictures from fixed samples of third-instar larvae lobe brains showing the immunofluorescence of the stress marker DAP and the Nb marker (DPN) at 24 and 48 hours after induction of aneuploidy. (E) Quantification of relative DAP-positive signal per lobe area from 24 to 48 hours AHS tissues. Brat mutant lobe brains showed a clear reduction in the presence of the aneuploidy-induced stress marker DAP at 24 hours AHS. *n* = number of lobe brains. ****P < 0.0001. Scale bar = 40 μm. Individual numerical values for the presented graphs can be found in S1 Data. AEL, after egg laying; AHS, after heat-shock induction; Brat, Brain tumor; DAP, Dacapo; DPN, Deadpan; HS, heat shock; Nb, Neuroblast; z-proj, z projection.

We took advantage of system to acutely induce aneuploidy and examine whether abnormal karyotypes trigger a stress response in the developing *Drosophila* brain and, if so, what the kinetics of such response is. We assessed, by immunohistochemistry, the presence of P53 and the senescence marker Dacapo (DAP, a p21/p27 homologue [45,46]) after the loss of cohesin and consequent aneuploidy. We observe that both P53 and the DAP accumulation start to be evident at 12 hours AHS, but only at 24 hours AHS are a significant number of different cell types labeled with these markers observed (Fig 6A, 6B and 6C). Furthermore, the large majority of cells that appeared positive for these markers are not Nb-like cells (mainly based on size and shape of the staining, Fig 6A). Nb-like cells stained with these markers are noticeable only at 48 hours AHS (Fig 6A, arrowheads and dashed circles), suggesting that despite their aneuploid state, neural stem cells are delayed at displaying an evident stress response. We confirmed this observation by quantifying specifically the appearance of cells costained with these markers (P53 and DAP) and the Nb marker DPN through time (S6A, S6B and S6C Fig). In addition to mitotic fidelity, cohesin has also been implicated in DNA damage response [47] and recent studies show that silencing RAD21 leads to the accumulation of gamma histone H2A X variant (γH2AX) foci [48]. Although it is impossible to dissociate mitotic loss of cohesin and the ensuing mitotic defects, abnormal karyotypes, and DNA damage, we reasoned that the ability of our tool to restore cohesin function in the next G1/S-phase would minimize the effect of RAD21 depletion on promoting double strand breaks (DSBs). To test this, we compared the presence of Histone H2A variant H2Av foci (H2AX homolog in *Drosophila*) after induced RAD21 depletion with and without restoring RAD21 function. Consistent with our hypothesis, restoring RAD21 activity hours after its depletion keeps the amount of H2Av foci not significantly different from the controls. Yet, if depletion of RAD21 is long-term, a substantial increase in the number of H2Av foci is observed at 8 and 12 hours AHS (S7A and S7B Fig). Although we cannot exclude DNA damage (or even the presence of micronuclei; see S5A and S5B Fig) as a contributing factor to the stress response observed in the tissue, it does not seem sufficient to explain the degree of Nb loss and stress markers observed in the tissue mainly at 24 hours AHS.

### Forced self-renewal delays the stress response

The delayed stress response (i.e., approximately 48 hours after induction of aneuploidy) in the neural stem-cell pool may imply a selective aneuploidy tolerance of Nbs when compared to the other cell types of the developing brain. To investigate this possibility, we took advantage of the Brain tumor (*brat*) mutant condition [49]. In *brat* mutant larvae brains, each Nb divides into two daughter cells that retain Nb-like properties as they continue self-renewing, leading to the formation of a tumor-like neoplasm [50,51]. We reasoned that if indeed Nbs are more resistant to aneuploidy, the complete occupancy of the developing brain by Nb-like cells observed in the *brat* mutant phenotype should be sufficient to prevent the stress response observed at 24 hours AHS. To test this, we combined our system for acute induction of aneuploidy with *brat* mutations and analyzed the presence of stress markers at 24 and 48 hours AHS. In *brat* mutants, the Nb marker DPN stains almost all the cells in the brain, demonstrating the Nb-like state of the entire tissue (Fig 6D and 6E). As predicted, DAP appearance was significantly delayed in aneuploid *brat* mutants when compared to aneuploid brains alone (Fig 6D and 6E). The same result is observed for P53 staining (S8A and S8B Fig). These results suggest that Nbs are uniquely resistant to aneuploidy-associated stresses. Such a delayed response has the drawback that it enables continuous proliferation, as reported in our data and previous studies [37,38]. The continued proliferation, in turn, allows for further brain growth despite the presence of aneuploidy. Indeed, our results show that induced aneuploidy during larval development has no significant impact in the length of the brain and optic lobes in adult flies (S9A–S9D Fig), unlike in chronic disruption states that result in microcephaly [13,38].

### Protecting only the developing brain from induced aneuploidy rescues adult life span

Upon aneuploidy challenge, we observed a striking difference across analyzed *Drosophila* tissues: whereas epithelial tissues like wing discs are able to regenerate from this injury (S2A and S2B Fig), about half of neural stem cells are lost, while the remaining half continues proliferating and becomes highly chromosomally unstable (Fig 4A and 4E). These findings, together with the fact that flies that survive the developmental aneuploidy induction show severe motor defects in otherwise healthy adult morphology, led us to hypothesize that the brain is the only tissue restricting aneuploid fly development.

**Fig 7:**
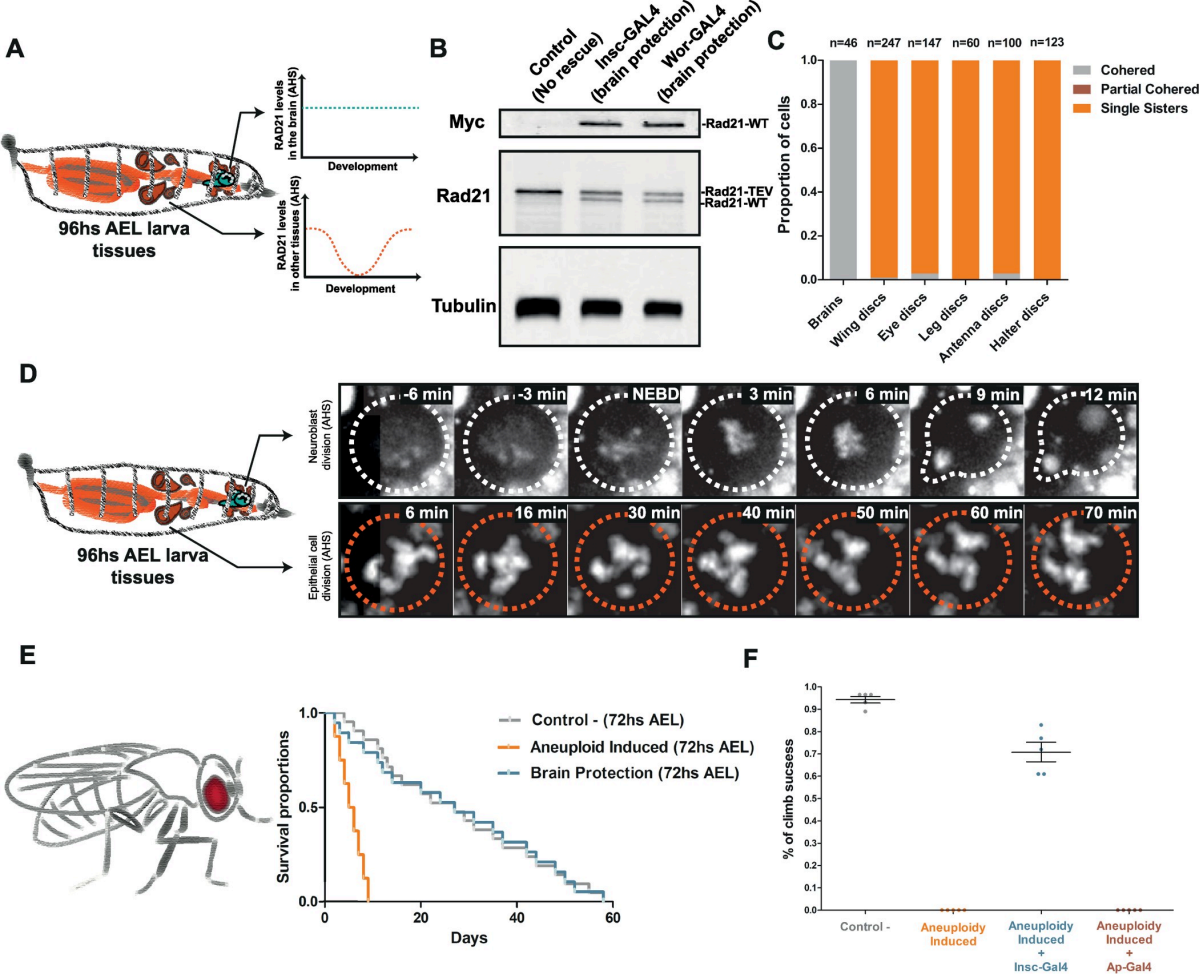
Protecting only the developing brain from induced aneuploidy rescues the adult life span. (A) Graphic scheme depicting how the developing brain is protected from the loss of cohesin and induction of aneuploidy upon the constitutive expression of the RAD21-WT driven by Nb-specific Gal4 (*Insc-Gal4* or *Wor-Gal4*). In contrast, the rest of the dividing tissues from the larva experience the acute inactivation of cohesin complex (by TEV cleavage of RAD21-TEV) AHS. (B) Western blots showing the production of both cleavable RAD21-TEV and RAD21-WT, which is resistant to TEV cleavage, in third-instar brains. *Insc-Gal4* and *Wor-Gal4* drivers result in expression of RAD21-WT in the third-instar brain before the heat shock. (C) Quantification of cohesive states of third-instar larvae Nbs and epithelial cells from different imaginal discs following heat shock. *Insc-Gal4* protects the brain from cohesin loss but has no effect in the imaginal discs. (D) Live-imaging cohesin profiles of Nbs and epithelial cells during mitotic divisions after the heat-shock. *Insc-Gal4* prevents mitotic delay caused by cohesin loss in the Nbs. (E–F) (E) Kaplan–Meier survival curves showing fractional survival as a function of time. Protection of the brain tissue from the induced aneuploidy rescues the adult life span of the ecloded flies with 72 hours AEL heat shock. (F) Climbing assay of adult flies. Percentage of climb success was plotted over the halfway point (10 cm). Protection of the brain tissue from induced aneuploidy by using Insc-GAL4 rescues motor defects of the ecloded flies with the 72 hours AEL heat shock. Contrastingly, protection of the wing and haltere disc epithelial tissues by using apterous-GAL4 does not rescue the motor defects. Individual numerical values for the presented graphs can be found in S1 Data. AEL, after egg laying; AHS, after heat-shock induction; GAL4, yeast transcription activator protein Gal4; *insc*, *inscutable*; myc, protein tag derived from the c-myc gene product; Nb, Neuroblast; NEBD, Nuclear Envelope Breakdown; RAD21, Double-strand-break repair protein rad21 homolog; TEV, Tobacco Etch Virus; *wor*, *worniu*; WT, wild type.

To test this hypothesis, we devised a system to protect the brain from cohesin removal and consequent aneuploidy. To achieve this, we complemented our reversible cohesin cleavage system with brain-specific expression of RAD21-WT throughout the course of the experiment (Fig 7A). In this way, TEV presence should lead to cohesin loss in all larval tissues that survive solely on RAD21-TEV at the time of heat shock. In contrast, neural stem cells should be resistant to this challenge because they express both RAD21-TEV and RAD21-WT (Fig 7B). Nb-specific expression of RAD21-WT was achieved by the use of *inscutable-Gal4* (*insc-Gal4*) or *worniu-Gal4* (*wor-Gal4*) drivers to constitutively express *UAS-Rad21-wt-myc* in the developing brain (Fig 7B). As expected, constitutive presence of RAD21-WT in the brain prevents any cohesin defects in third-instar larvae Nbs (Fig 7C). To confirm that the rescue of sister chromatid cohesion occurs exclusively in the brain, we performed parallel characterization of the first mitotic division AHS in multiple imaginal discs derived from the same larvae. As anticipated, full cohesin cleavage was observed in all the dividing epithelial tissues (Fig 7C and 7D). Notably, protecting only the brain from developmental aneuploidy fully rescued the severe motor defects of the ecloded flies from the 72 hours AEL heat-shock, as demonstrated by mobility essays (Fig 7F and S6 Movie). The brain protection was enough to rescue the life span of approximately 70% of the adult flies affected by organism-wide aneuploidy during development, demonstrating that the brain is indeed the most sensitive tissue when challenged with aneuploidy (Fig 7E).

## Discussion

### Reversible disruption of mitotic fidelity enables tracing of aneuploidy

We developed a novel genetic tool in *Drosophila* to study aneuploidy in vivo. This tool enables the induction of a controlled pulse of aneuploidy at the developmental stage of choice. The outcomes of using reversible perturbation are significantly different from the ones resulting from chronic disruption of mitotic fidelity. The long-term survival after aneuploidy challenge coupled with the reversibility of the mitotic perturbation overcomes one of the major limitations present in other metazoan models: we were able the study the kinetics of aneuploidy and its consequences across different tissues/developmental stages.

Cohesin loss and induction of aneuploidy is tolerated better by the organism if induced early in development, as observed by comparing the rates of eclosion. The developing larvae are progressively scaling mitotic machines, with each consecutive stage containing more divisions than the previous one [36]. This implies that the heat shock at the first- and third-instar larvae are not the same because they affect different number of dividing cells, thus generating different numbers of aneuploid progeny. Although the more parsimonious explanation for aneuploidy tolerance in early development would be a quantitative one, it is also important to mention that a developmental delay is observed after aneuploidy induction (e.g., delayed pupariation stage). It is well known that delayed development allows the organism to adjust their growth programs after disturbances [52,53]. This induced delay is a development-stage dependent response because some perturbations only appear to retard pupariation when induced at or before a certain stage in larval development, as, e.g., beginning of the third instar [54–56].

### Chromosome mis-segregation in Nbs leads to a complex array of karyotypes and cellular abnormalities

Nbs have been used as a system to study aneuploidy response in previous studies [11,35,38,57]. Recent studies postulate two different but not mutually exclusive mechanisms of response to induced aneuploidy: premature differentiation [35] and cell death by apoptosis [11]. We reasoned that if these are the only mechanisms of response to aneuploidy in neural stem cells, they should be detectable in high frequency after the aneuploidy induction by our acute approach. Contrary to that notion, both premature differentiation and cell death were detected at a very low frequency, even days after cells became aneuploid. It is important to note that the degree of aneuploidy in the Nbs upon cohesin loss should be around 98% because of the extensive genome shuffling prior to mitotic exit. Therefore, the finding that aneuploidy does not eliminate the entire Nb population strongly argues against the existence of specific, active mechanisms controlling the integrity of the neural stem-cell genome. The more plausible explanation is that the Nb elimination due to aneuploidy stems from a wide spectrum of abnormalities due to a randomized genome, documented throughout this study. Supporting this idea, recent studies in yeast have shown that the same aneuploid karyotypes can have different outcomes [58]

Examination of Nbs in real time after aneuploidy induction further revealed that aneuploidy is sufficient to induce chromosomal instability within a short time period (approximately 16 hours), which was shown in yeast and tissue culture but never in a metazoan system [41,42]. The appearance of chromosomal instability, characterized by a wide range of mitotic defects, takes several cell cycles after cohesin has been restored, which strongly supports the notion that chromosomal instability is a consequence of the abnormal karyotype and not the mitotic disruption initially applied. Overall, we observe a selection towards the accumulation of chromosomes, generating huge Nbs that keep proliferating despite their increased ploidy [35,38]. This clearly demonstrates that just a single round of chromosome mis-segregation in these cells is enough to originate a complex array of karyotypes, which can lead to mitotic abnormalities.

### Nb-like self-renewal confers resistance to aneuploidy-associated stresses

During the last years, studies in tissue culture and yeast cells have collected solid evidence on how aneuploid karyotypes can alter physiology of eukaryotic cells (reviewed in [59]). They can lead to different aneuploidy-associated responses that include oxidative, metabolic, replication, and proteotoxic stress, which likely contribute to p53 activation and, in most cases, cell senescence and/or cell death [43]. However, our understanding of how aneuploidy-induced stress at the cellular level influences development of tissues is very limited.

Our time-course assessment of classical stress response marker P53 and the *Drosophila* senescence marker DAP [45,46] clearly showed that the response to induced aneuploidy is not immediate and takes several hours (12 to 24 hours AHS). This delayed stress response is in agreement with recent observations in tissue culture, in which it has been shown that chromosome mis-segregation did not lead to the arrest in the following G1 in the vast majority of aneuploid daughter cells [60,61].

Our results highlight that cell identity might determine the kinetics of this stress response. Aneuploidy response is specifically delayed in the neural stem-cell pool (displayed mainly at approximately 48 hours AHS) compared to the rest of the tissue, which exhibits it considerably earlier. Forcing cells to self-renew using Brat mutations is sufficient to delay the appearance of P53 and DAP markers in the entire tissue, suggesting that uncontrolled self-renewal makes cells less sensitive to trigger a stress response. Quite fittingly, it was recently reported that aneuploidy in *Drosophila* Intestine Stem Cells (ISCs) results in increased stem-cell proliferation. Interestingly, ISCs do not activate apoptotic pathways in response to aneuploidy, suggesting that this type of cell is somewhat resistant to genomic imbalance [62]. Unusual resistance to altered ploidy was also observed in human and mouse embryonic stem cells (ESCs), mostly achieved by relaxing the cell-cycle control and uncoupling the spindle checkpoint from apoptosis [63]. The ability of neural stem cells to continue dividing despite the aneuploid karyotype dubbed them as aneuploidy “tolerant” [11]. Yet, based on our findings, it is clear that keeping these aneuploid cells is catastrophic for normal tissue architecture and development. Thus, aneuploidy may be “tolerated” better in Nbs, but the tissue as a whole is unable to be functional. In contrast, the “sensitivity” of epithelial cells enables the tissue to clean up and regrow properly.

### The developing brain restricts organism recovery after induced aneuploidy

Chromosomal aberrations have been long associated with neurological disorders [64]. However, their impact on brain development and function remains poorly understood, partially due to limitations of available experimental approaches. Previous studies in *Drosophila* have shown that the mitotic disruption in larvae Nbs generates a reduction of their brain size, reinforcing the idea about a link between aneuploidy and microcephaly [11,13,35,65]. However, our results showed that induced acute aneuploidy has no significant impact in the size of the adult brain. These findings suggest that the continued proliferation of neural stem cells, caused by incomplete cell elimination and delayed aneuploidy stress response, is sufficient to support the development of an apparently normal-sized organ. It is conceivable that the observed normal size reflects a sample selection because this analysis was restricted to flies that survived the aneuploid challenge (approximately 70%). Supporting this possibility, a screening performed to isolate anatomical brain mutants of *Drosophila* has shown that mutant strains showing altered brain shape and particularly small brains are very weak, having mostly the mutations that are lethal at pupa stage [66]. Despite the unaltered shape and size of the adult brains, we reasoned that the neural circuits are likely impaired in those brains, giving rise to the adult phenotype observed in all the surviving flies.

In accordance with the notion of the brain as the tissue most sensitive to aneuploidy, we show that preventing aneuploidy exclusively in the brain is sufficient to rescue the behavioral and life span defects promoted by developmental aneuploidy, suggesting that neural tissue is the most ill-equipped to deal with aneuploidy during development and imposes a significant cost for the organism. Several pathophysiological chromosomal disorders in humans—including trisomy 21, trisomy 18, and trisomy 13, as well as the mosaic disorder mosaic variegated aneuploidy (MVA, characterized by the presence of a different number of chromosomes in some cells)—are well known to result in intellectual disability [64], yet the impact of the aneuploid condition on brain development is still unclear [67,68]. Additionally, there is a lively debate about whether aneuploidy exists in metazoan brains and whether aneuploid neurons could be functional [69]. Usage of an inducible, in vivo system such as ours opens up the way for the exploration of this conundrum. Future work should also aim at elucidating the molecular mechanisms underlying the physiological changes in stem/somatic cells generated by aneuploidy and its implications on tissue development and homeostasis.

## Materials and methods

### Fly husbandry and genetics

Flies were raised using standard techniques at room temperature (20°C–22°C). All stocks used in this study are summarized in Table 1. We established both chronic and the acute inactivation of cohesin complex by crossing the following genotypes: *w;hspr-nlsV5TEV;Rad21(ex*^*3*^*)/TM6B* with *w;;tubpr-Rad21(550-3TEV)-EGFP*,*Rad21(ex*^*15*^*)*,*polyubiq-His-RFP* and *w;hspr-nlsV5TEV;Rad21(ex*^*3*^*)*,*hspr-Gal4*,*UAS-Rad21(wt)-myc/TM6B* with *w;;tubpr-Rad21(550-3TEV)-EGFP*,*Rad21(ex*^*15*^*)*,*polyubiq-His-RFP*, respectively. The progeny were then heat-shocked once at 37°C for 45 min at the desired developmental stage. The correct genotype larvae were selected based on the absence of the “tubby” phenotype; the heat-shocked “tubby” larvae were used as negative controls (control heat shock). As genetic control, we used the same genotypes for the induction of aneuploidy but without performing the heat shock.

**Table 1:** Stocks used in this study.

Stock Genotype	Reference
*w;hspr-nlsV5TEV;Rad21(ex*^*3*^*)/TM6B*	[24]
*w;;tubpr-Rad21(550-3TEV)-EGFP*,*Rad21(ex*^*15*^*)*,*polyubiq-His-RFP*	[70]
*w;hspr-nlsV5TEV;Rad21(ex*^*3*^*)*,*hspr-Gal4*,*UAS-Rad21(wt)-myc/TM6B*	This study
*w;hspr-nlsV5TEV*,*brat*^*TS*^*/CyO-CTG;tubpr-Rad21(550-3TEV)-EGFP*,*Rad21(ex*^*15*^*)*,*polyubiq-His-RFP*	This study
*w;brat*^*1*^*/CyO-CTG; Rad21(ex*^*3*^*)*,*hspr-Gal4*,*UAS-Rad21(wt)-myc/TM6B*	This study
*w;UAS-P35;tubpr-Rad21(550-3TEV)-EGFP3*,*Rad21(ex*^*15*^*)*,*polyubiq-His-RFP*	This study
*w;HisH2AvDmRFP1II*.*2/CyO;363*,*CGCIII*.*1(R26)/TM3*,*Ser*	[71]
*brat*^*ts1*^*rdo*^*1*^*hook*^*1*^*pr*^*1*^*/CyO*	BDSC #3991
*brat*^*1*^*rdo*^*1*^*hook*^*1*^*pr*^*1*^*/CyO*	BDSC #3988
*w;P[wor*.*GAL4*.*A]2;Dr*^*1*^*/TM3*,*P[Ubx-lacZ*.*w+]TM3*,*Sb*^*1*^	BDSC #56553
*w; P[GawB]*^*inscMz1407*^	BDSC #8751
*w;P{GawB}*^*apmd544*^*UAS-EGFP/CyO;Rad21(ex3)*,*hspr-Gal4*,*UAS-Rad21(wt)-myc/TM6B*	This study

**Abbreviations**: Brat, Brain tumor; Gal4, yeast transcription activator protein Gal4; myc, protein tag derived from the c-myc gene product; RAD21, Double-strand-break repair protein rad21 homolog; TEV, Tobacco Etch Virus; UAS, upstream activating sequence; WT, wild type.

To determine the proportion of adult eclosion, the crosses mentioned were raised in cages to monitor the time of egg collection. After 6 hours of collection, the plates were removed from the cages, the number of eggs was counted, and the plates were kept until larvae hatched. The plates were then heat-shocked at 37°C for 45 min at different larvae developmental time (approximately 48 hours AEL, approximately 72 hours AEL, approximately 96 hours AEL, and approximately 120 hours AEL (± 6 hours)) and placed in a new clean plastic cage. Once they reached pupae stage (“yellow body”), the pupae were gently removed with a wet brush and separated into “tubby” (control heat shock) and “no tubby” phenotypes (conditions). The different batches of pupae were placed over agar plates covered with two layers of absorbent paper to maintain the humidity and counted. The plates with the pupae were kept at room temperature until flies ecloded, and the proportion of eclosion was calculated.

To combine the induction of aneuploidy (acute cohesin inactivation) and the *brat* mutant genetic background, we generated the following stocks: *w;brat*^*1*^*/CyO-CTG;Rad21(ex*^*3*^*)*,*hspr-Gal4*,*UAS-Rad21(wt)-myc/TM6B* and w*;hspr-nlsV5TEV*,*brat*^*TS*^*/CyO-CTG;tubpr-Rad21(550-3TEV)-EGFP*, *Rad21(ex*^*15*^*)*,*polyubiq-His-RFP*. These stocks were crossed, the progeny were heat-shocked once at 37°C for 45 min at the developmental stage desired, and the genotype *w;brat*^*1*^*/hspr-nlsV5TEV*,*brat*^*TS*^*;Rad21(ex*^*3*^*)*,*hspr-Gal4*,*UAS-Rad21(wt)-myc/tubpr-Rad21(550-3TEV)-EGFP*,*Rad21(ex*^*15*^*)*,*polyubiq-His-RFP* was selected at larva stage based on the absent of both GFP signal and “tubby” phenotype.

To inhibit apoptosis, we induced the overexpression of the baculovirus p35 in the context of the genetic background for acute inactivation of cohesin complex. To achieve this purpose, we generated the following stock: *w;UAS-P35;tubpr-Rad21(550-3TEV)-EGFP3*,*Rad21(ex*^*15*^*)*,*polyubiq-His-RFP* to be crossed with *w;hspr-nlsV5TEV;Rad21(ex*^*3*^*)*,*hspr-Gal4*,*UAS-Rad21(wt)-myc/TM6B*. The progeny were then heat-shocked once at 37°C for 45 min at the developmental stage desired.

Finally, for the “brain rescue” experimental setup, we generated the following stocks: *w;insc-Gal4;tubpr-Rad21(550-3TEV)-EGFP*,*Rad21(ex*^*15*^*)*,*polyubiqpr-His-RFP* and *w;wor-Gal4;tubpr-Rad21(550-3TEV)-EGFP*,*Rad21(ex*^*15*^*)*,*polyubiqpr-His-RFP*. These stocks were crossed with the *w;hspr-nlsV5TEV;Rad21(ex*^*3*^*)*,*hspr-Gal4*,*UAS-Rad21(wt)-myc/TM6B* stock. The crosses and the progeny were raised and treated as described above for the determination of the eclosion proportion.

### Life span analysis

Life span was measured at room temperature according to standard protocols. In brief, newly ecloded animals (0 to 3 days) were collected (50 per genotype: “control,” “Aneuploidy,” and “Aneuploidy + brain rescue”) and then placed in vials (up to 10 per vial) and transferred to fresh vials every two days. Survival was recorded for each vial. Because of the reduced mobility of the aneuploidy genotypes, we scored flies stacked in the food as death events in all the vials analyzed. We created survival curves with Prism 5.00 for Windows (GraphPad Software, San Diego, CA, USA) using the method of Kaplan and Meier.

### Climbing assay

For the climbing assay, flies were anesthetized with CO_2_, separated into groups of around 20 adults (3 replicas for each genotype), and allowed to recover for 2 hours before being subjected to a climbing assay. Briefly, the groups of over 20 flies were placed in an empty climbing vial and then tapped down to the bottom. They were allowed to climb past the halfway point from the bottom of the vial for 30 seconds (10 cm). The number of flies above the 10 cm mark was recorded as the percentage of flies able to climb.

### Histology

Briefly, flies were anesthetized with CO_2_ and then were placed gently in agarose blocks to immobilize them and prevent any damage to the head or eyes. The agarose blocks with the flies were immersed in Carnoy fixative overnight at 4°C. The next day, the Carnoy solution was removed, and three 70% ethanol washes were performed. Immediately after, the flies were decapitated, and the heads were oriented one by one in melted 2% agarose to guarantee similar orientation of the tissue sections. Agarose blocks were then processed and embedded, and the whole head was sectioned into 5-μm-thick sequential sections and stained with hematoxylin–eosin. The histology was performed in the Histopathology unit at Instituto Gulbenkian de Ciência, and the slides were analyzed by a pathologist with a DMLB2 microscope (Leica, Wetzlar, Germany). Images were acquired with a DFC320 camera (Leica) and NanoZoomer-SQ Digital slide scanner (Hamamatsu, Hamamatsu City, Japan).

### Live-cell imaging

Larvae third-instar brains were dissected in Schneider medium supplemented with 10% FBS, and intact brains were mounted on a glass-bottomed dish (MatTek In Vitro Life Science Laboratories, Bratislava, Slovak Republic), covered with an oxygen-permeable membrane (Xylem Analytics UK, Tunbridge Wells, United Kingdom), and sealed with Voltalef oil 10S (VWR, Radnor, PA, USA). This procedure allowed long-term imaging of brains for periods of up to 10 hours.

For imaging of imaginal discs and early instar larvae brains, tissues were dissected in Schneider medium with 10% FBS. Dissected discs were placed and oriented in a 200 μl drop of medium at the bottom of a glass-bottomed dish (MatTek).

Live imaging was performed on a spinning disc confocal using a Revolution XD microscope (Andor, Belfast, UK) equipped with a 60× glycerol-immersion 1.30 NA objective (Leica Microsystems) and an iXon Ultra 888 1,024 × 1,024 EMCCD (Andor). 25–35 Z-series optical sections were acquired 0.5–1 μm apart.

### Brain spreads and immunofluorescence

For brain spreads and immunofluorescence, third-instar larvae brains were dissected in PBS, incubated with 100 μM colchicine for one hour, hypotonic shocked in 0.5% sodium citrate for 2–3 min, and fixed on a 5-μl drop of fixative (3.7% formaldehyde, 0.1% Triton-X100 in PBS) placed on top of a siliconized coverslip. After 30 seconds, the brains were squashed between the coverslip and a slide, allowed to fix for an additional 1 min, and then placed in liquid nitrogen. Slides were further extracted with 0.1% Triton X-100 in PBS for 10 min and used for immunofluorescence following standard protocols. Primary antibodies were rat anti-CID (gift from Claudio E. Sunkel) used at 1:2,000; cleaved *Drosophila* Dcp-1 (Asp216) antibody (1:300) #1679578S (Cell Signaling Technology, Danvers, MA, USA); CC3 (Asp175) antibody #9661 (1:300) (Cell Signaling Technology); anti-DPN antibody #ab195173 (1:1,500) (Abcam, Cambridge, UK); anti-P53 (1:150) p53 25F4, which was deposited to the DSHB by G. M. Rubin (DSHB Hybridoma Product p53 25F4); anti-histone H2AvD pS137 rabbit (1:1,000) (Rockland, Limerick, PA, USA); anti-lamin (1:1,000) ADL84.12, which was deposited to the DSHB by P. A. Fisher (DSHB Hybridoma Product ADL84.12); and anti-DAP (1:50) NP1, which was deposited to the DSHB by I. Hariharan (DSHB Hybridoma Product NP1).

Secondary antibodies conjugated with fluorescent dyes from Alexa series (Invitrogen, Carlsbad, CA, USA) were used according to the manufacturer's instructions.

Third-instar wing imaginal disc fixation and staining, as well as immunofluorescence of whole brains, was performed using standard procedures [72]. Briefly, third-instar larvae wing disc tissue (still attached to the larva body) was fixed on ice for 30 min. The fixative consisted of 4% formaldehyde (Polysciences, Warrington, PA, USA) in 1× PEM buffer solution. Following this, tissues were washed by gentle agitation three times for 20 min in 1× PBS + 0.1% Triton X-100 (PBS-T). Primary antibody incubation was performed overnight at 4 °C in PBS-T supplemented with 1% BSA and 1% donkey serum. The following day, the tissues were washed again and incubated for 2 hours at room temperature with the appropriate secondary antibodies diluted in PBS-T solution. Finally, after the wash of secondary antibodies, wing discs were mounted in Vectashield (Vector Laboratories, Burlingame, CA, USA). Fluorescence images were acquired with a ×40 HCX PL APO CS oil immersion objective (numerical aperture: 1.25–0.75) on a Leica SP5 confocal microscope.

### FISH

Brains from third-instar larvae were dissected in PBS, incubated with 100 μM colchicine for one hour, and transferred to 0.5% sodium citrate solution for 3–4 min. Then, the brains were transferred to a fixative containing 11:11:2 methanol/acetic acid/MQ water for 30 seconds before being placed in a droplet of 45% acetic acid for 2 min, squashed, and transferred to liquid nitrogen. Then, the coverslip was removed and the slide incubated in absolute ethanol for 10 min at −20 °C (freezer incubation). The slides were air dried at 4 °C (20 min). The slides were dehydrated at room temperature in 70%, 90%, and absolute ethanol for 3 min prior to DNA denaturation in 70% formamide–2× SCC solution for 2 min at 70 °C. This was done on a thermomixer set at 70°C with a formamide solution heated to 70 °C. Then, the slides were transferred to cold 70% ethanol (−20 °C) and dehydrated at room temperature in 90% and absolute ethanol for 3 min. FISH probes were denatured in the hybridization buffer at 92°C for 3 min. Hybridization was done overnight at 37 °C using 30 μl of FISH hybridization buffer/probe mix per slide. Hybridization buffer: 20% dextran sulfate in 2× SCCT/50% formamide/0.5 mg/ml salmon sperm DNA. Then, slides were washed 3 × 5 min in 50% formamide–2× SCC at 42 °C and 3 × 5 min in 0.1× SCC at 60 °C. These steps were done on the thermomixer, with the solutions previously heated to desired temperatures. Finally, the slides were washed in PBS and mounted in Vecta shield with DAPI. The probes were used in the final concentration of 70 Nm in hybridization buffer. Probes used were Chr_X (359-bp satellite DNA) A546-GGGATCGTTAGCACTGGTAATTAGCTGC and Ch_3 (dodeca satellite DNA) Cy5-ACGGGACCAGTACGG DNA probes, Chr_2 A488-(AACAC).

### Western blot

To analyze RAD21 protein amounts, *Drosophila* tissues were dissected in PBS and homogenized with a pestle in sample buffer. Samples were centrifuged and boiled for 5 min in 2× sample buffer. Samples were loaded on a 13% SDS-gel for electrophoresis and then transferred to nitrocellulose membranes. Western blot analysis was performed according to standard protocols using the following antibodies: anti-α-tubulin (1:50,000, DM1A, Sigma-Aldrich Cat# T9026; Sigma-Aldrich, St. Louis, MO, USA), guinea pig anti-Rad21 [73], and V5 Tag mouse monoclonal antibody (Novex, Thermo Fisher Scientific, Waltham, MA, USA).

### Image analysis

Imaging analysis was performed using FIJI software [74]. For z projections, slices were stacked into maximum intensity (10 frames, 2 μm each). Some pictures were rotated and/or flipped to orient them in the same way.

### Statistical analysis

Statistical analysis and graphic representations were performed using Prism 5.00 for Windows (GraphPad Software, San Diego, CA, USA). Unpaired *t* test or one-way ANOVA (using the Bonferroni’s multiple comparison) were applied depending on the measurements analyzed in the corresponding experiment. Sample size details are included in the respective plotted graphs.

## Supporting information

S1 Fig Heat-shock treatment induces brain aneuploidy at all stages of development. (A–C) Stills from live imaging of lobe brains at different larvae stages (48, 72, and 96 hours AEL); dashed circles are highlighting the Nbs in the lobes (*N* > 3 brains per condition). The number of dividing Nbs increases with larvae development. Cohesive state of Nbs after the loss of cohesin and subsequent rescue in 48, 72, and 96 hours AEL larvae were plotted. (D) Western blot of RAD21 cleavage and rescue dynamics in 72-hours–AEL larvae brains (over 10 western blots were performed to validate the system). Individual numerical values for the presented graphs can be found in S2 Data. AEL, after egg laying; NB, Neuroblast; RAD21, Double-strand-break repair protein rad21 homolog.
(PDF)

S2 Fig Epithelial tissues recover from high levels of aneuploidy by cell death and compensatory proliferation. (A–B) (A) Reversible cohesin cleavage results in apoptosis in the third-instar wing discs (dashed shapes depict the wing disc areas). The amount of apoptosis per disc was measured by area of CC3 immunofluorescence at 24, 48, and 72 hours AHS. (B) Rescue of cohesin function significantly reduced the amount of apoptosis within 48 hours AHS. In contrast, chronic inactivation of cohesin complex (no cohesin rescue) displayed high levels of apoptosis through time. Control− (Control HS); Control+ (Irradiation: 4,000 rads). *P < 0.05; ****P < 0.0001. Scale bar = 40 μm. Individual numerical values for the presented graphs can be found in S2 Data. AHS, after heat-shock induction; AI, After Irradiation; BHS, Before Heat-Shock; BI, Before Irradiation; CC3, Cleaved Caspase 3; HS, heat shock; z-proj, z projection.
(PDF)

S3 Fig RAD21 cleavage and rescue induces loss of cohesin in all examined dividing tissues. (A) Stills from live imaging of leg, eye, antennae, and haltere third-instar imaginal discs after induction of RAD21 cleavage. Dashed squares display epithelial cells from the imaginal discs undergoing mitosis with loss of cohesin (see enlarged picture). (B) The cell-cycle profile evaluation of the third-instar control wing disc with or without the heat shock, using the fly FUCCI system. The high incidence of cells affected by reversible cohesin cleavage is consistent with a high frequency of cells in G2/M in this tissue (see Merge). GFP: G1 cells; RFP: S-phase cells; Merge: G2/M Cells (*n* > 500, at least three wing discs analyzed). Individual numerical values for the presented graphs can be found in S2 Data. FUCCI, Fluorescence Ubiquitination Cell Cycle Indicator; GFP, green fluorescent protein; G2, Gap 2 phase; M, Mitosis; RAD21, Double-strand-break repair protein rad21 homolog; RFP, red fluorescent protein; S, Synthesis phase.
(PDF)

S4 Fig Aneuploidy results in low frequency of stem identity loss and cell death in Nbs. (A–B) (A) Pictures from fixed samples of third-instar larvae lobe brains stained with DPN, Pros, and Histone RFP (DNA). Induction of aneuploidy results in the loss of stem-cell identity measured by the absence of DPN (stem-cell marker, white arrowhead with dashed circle), appearance of Pros (differentiation marker, yellow arrowhead with dashed circle), or both markers together in cell nucleus with “Nbs-like shape.” (B) Percentage of loss of stem-cell identity in the neural stem-cell pool at different time points after the induction of aneuploidy. These events are observed at very low frequency. *n* = number of Nb-like cells. Scale bar = 40 μm. (C–E) (C) Pictures from fixed samples of third-instar larvae lobe brains stained with DPN, CC3 (death marker), DCP1 (death marker), and rhodamine phalloidin (Actin). Induction of aneuploidy results in cell death measured by the presence of CC3 or DCP1 signals (white arrowheads with dashed circles) in cells with “Nbs-like shape.” (D and E) Quantification of cell death signals CC3 and DCP1 per larvae brain lobes at 24 hours AHS. The presence of positive signal for the cell death markers in Nb-like cells is very low. **P < 0.01. Scale bar = 40 μm. (F) Quantification of Nbs at the CB in third-instar lobe brains assessed by immunofluorescence with the Nb marker DPN. Inhibition of apoptosis by overexpression of baculovirus P35 does not rescue Nb number after 24-hours–induced aneuploidy. *n* = number of lobe brains. ****P < 0.0001. Individual numerical values for the presented graphs can be found in S2 Data. AHS, after heat-shock induction; CB, central brain; CC3, Cleaved Caspase 3; DCP1, Death Caspase-1; DPN, Deadpan; Nb, Neuroblast ns, not significant; Pros, Prospero; RFP, red fluorescent protein.
(PDF)

S5 Fig Analysis of micronuclei formation after reversible loss of cohesin. (A–B) (A) Micronuclei assessment upon aneuploidy induction. Only after 24 hours AHS was the percentage of micronuclei per Nb counts different from the control (Control HS: 5% versus aneuploidy-induced 24 hours AHS: 24%). (B) Quantification of micronuclei at the different conditions. Micronuclei were assessed by counting DNA signal (green) together with Lamin immunofluorescence (red) in spreads from brain tissues at 8 and 24 hours AHS. Micronuclei were defined as a DNA particle with enclosed-by-LAMIN staining with a perimeter (Fiji measurement) smaller than 60. Number of brains analyzed (Control HS (8 + 24 hours AHS) = 10; 8 hours AHS = 8; 24 hours AHS = 8). *n* = number of cells. Micronuclei are indicated by white dashed circles with arrowhead. Aneuploid Nbs are indicated by yellow circles with arrowhead. High magnification of micronuclei is shown by dashed squares. Individual numerical values for the presented graphs can be found in S2 Data. AHS, after heat-shock induction; HS, heat shock; Nb, Neuroblast.
(PDF)

S6 Fig Aneuploidy-induced stress response is particularly delayed in the Nbs. (A–C) Pictures from fixed samples of third-instar larvae lobe brains showing the immunofluorescence of canonical stress-response markers P53 and DAP together with the Nb marker DPN at 48 hours AHS. Nbs display a delayed aneuploidy stress response at 48 hours AHS (arrowheads with dashed circles). (B and C) Quantification of the kinetics of the aneuploidy-induced stress response at 6, 12, 24, and 48 hours AHS in Nbs (DPN+). Scale bar = 40 μm. Individual numerical values for the presented graphs can be found in S2 Data. AHS, after heat-shock induction; DAP, Dacapo; DPN, Deadpan; Nb, Neuroblast.
(PDF)

S7 Fig Analysis of DNA damage after reversible loss of cohesin. (A–B) (A) Pictures from fixed samples of third-instar larvae lobe brains showing the immunofluorescence stainings with the H2Av antibody (DSBs marker in *Drosophila*) at 4, 8, 12, 24, and 48 hours with and without rescuing RAD21 depletion. (B) Quantification of the H2Av signal kinetics after loss of cohesin and consequent aneuploidy at 4, 8, 12, 24, and 48 hours AHS. ***P < 0.001; ****P < 0.0001 compared with no-HS control. Individual numerical values for the presented graphs can be found in S2 Data. AHS, after heat-shock induction; DSB, double strand break; HS, heat shock; H2Av, Histone H2A variant; RAD21, Double-strand-break repair protein rad21 homolog.
(PDF)

S8 Fig Aneuploidy-induced P53 accumulation is delayed in the neural stem-cell pool. (A–B) (A) Brat mutant lobe brains showed a clear reduction in the presence of the aneuploidy-induced stress marker P53 at 24 hours AHS. (B) Quantification of relative P53-positive signal per lobe area from 24 to 48 hours AHS. *n* = number of lobe brains. ****P < 0.0001. Scale bar = 40 μm. Individual numerical values for the presented graphs can be found in S2 Data. AHS, after heat-shock induction; Brat, Brain tumor; z-proj, z projection.
(PDF)

S9 Fig Adult brains do not show any major alteration in size after acute induction of aneuploidy during development. (A–C) (A) Dissected brains of adult flies from control and developmental aneuploidy-induced (72-hours–AEL heat shock) organisms. (B and C) Quantifications of lobe diameter and brain length in control and developmental aneuploidy-induced (72- and 96-hours–AEL heat shock) adult flies showed no significant differences. *n* = number of brains. (D) Histology analysis of brains from control and aneuploidy-induced-during–development (72-hours–AEL heat shock) adult flies, 1 day after eclosion. Frontal sections at approximately midbrain showed no signal of neurodegenerative process (vacuolization). Individual numerical values for the presented graphs can be found in S2 Data. AEL, after egg laying; HE, hematoxylin–eosin; ns, not significant.
(PDF)

S1 Movie Demonstration of cohesin cleavage and rescue system in a single Nb after the heat shock. Movie depicting live imaging of the first and second mitosis after the heat chock, in a single third-instar larvae Nb; imaging of H2AvD mRFP1 in 3-min timeframes. First division after the heat shock results in complete cohesin loss, while the second provides a full cohesin rescue. H2AvD, Histone H2A variant Drosophila; Nb, Neuroblast; RFP, red fluorescent protein.
(MOV)

S2 Movie Demonstration of cohesin cleavage and rescue in the entire larval brain lobe after the heat shock. Movie depicting live imaging of the third-instar larvae brain lobe; imaging of HisH2AvD mRFP1 in 3-min timeframes. First divisions after the heat shock result in complete cohesin loss and genome shuffling, consecutive divisions result in cohesin rescue. His2AvD, Histone H2A variant Drosophila; RFP, red fluorescent protein.
(MOV)

S3 Movie Cohesin loss and rescue results in eclosion of adults with motion defects, despite healthy wing and appendage morphology. Movie depicting live imaging of the adults eclosed after the aneuploidy challenge at the age of 72 hours AEL. AEL, after egg laying.
(MOV)

S4 Movie A climbing assay comparing heat shock controls and flies challenged with aneuploidy during development. (MOV)

S5 Movie 48 hours AEL heat shock results in eclosion of adults with lethargic behavior despite healthy wing and appendage morphology. Movie depicting live imaging of the adult eclosed after the aneuploidy challenge at the age of 48 hours AEL. AEL, after egg laying.
(MOV)

S6 Movie Climbing assay comparing brain-rescued flies and flies challenged with aneuploidy during development. (MOV)

S1 Data Individual numerical values that underlie the summary data displayed in the Main Figures. (PZF)

S2 Data Individual numerical values that underlie the summary data displayed in the Supplementary Figures. (PZF)

## Abbreviations

AEL: after egg laying
AHS: after heat-shock induction
Brat: Brain tumor
CB: central brain
CC3: Cleaved Caspase 3
CID: Histone H3-like centromeric protein
DAP: Dacapo
DCP1: Death Caspase-1
DPN: Deadpan
DSB: double strand break
ESC: embryonic stem cell
FISH: Fluorescence In Situ Hybridization
FUCCI: Fluorescence Ubiquitination Cell Cycle Indicator
GAL4: yeast transcription activator protein Gal4
G2: Gap 2 phase
HS: heat shock
H2Av: Histone H2A variant
*insc*: *inscrutable*
HSprom: HS promoter
*hspr-nlsV5TEV*: heat shock promoter-nuclear localization signal V5 epitope Tobacco Etch Virus
ISC: Intestine Stem Cell
M: Mitosis
MVA: mosaic variegated aneuploidy
*myc*: protein tag derived from the c-myc gene product
Nb: Neuroblast
ns: not significant
PBS-T: 1× PBS + 0.1% Triton X-100
Pros: Prospero
RAD21: Double-strand-break repair protein rad21 homolog
S: Synthesis phase
SAC: Spindle Assembly Checkpoint
SMC: Structural Maintenance of Chromosome
TEV: Tobacco Etch VirusUAS, upstream activating sequence
*wor*: *worniu*
WT: wild type
z-proj: z projection
γH2AX: gamma histone H2A X variant
