# Supplementary figures and images for: Induced aneuploidy in neural stem cells triggers a delayed stress response and impairs adult life span in flies
Source: PLoS Biol. 2019 Feb 22;17(2):e3000016. doi: 10.1371/journal.pbio.3000016 (PMC6402706; doi:10.1371/journal.pbio.3000016)

# Supp Figure 1

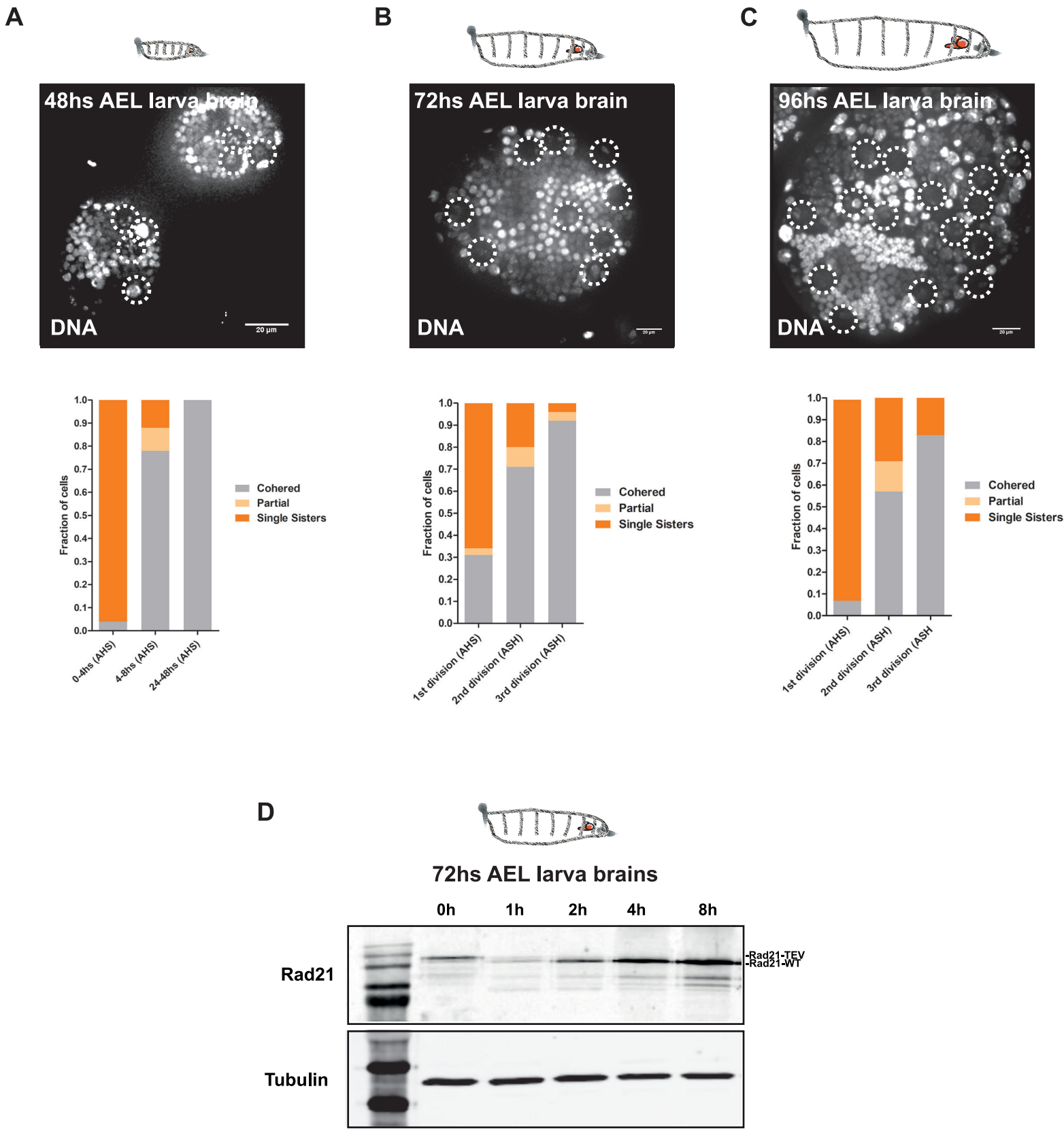

Supplement: S1 Fig — (A–C) Stills from live imaging of lobe brains at different larvae stages (48, 72, and 96 hours AEL); dashed circles are highlighting the Nbs in the lobes (N > 3 brains per condition). The number of dividing Nbs increases with larvae development. Cohesive state of Nbs after the loss of cohesin and subsequent rescue in 48, 72, and 96 hours AEL larvae were plotted. (D) Western blot of RAD21 cleavage and rescue dynamics in 72-hours–AEL larvae brains (over 10 western blots were performed to validate the system). Individual numerical values for the presented graphs can be found in S2 Data. AEL, after egg laying; NB, Neuroblast; RAD21, Double-strand-break repair protein rad21 homolog. (PDF) [file pbio.3000016.s001.pdf]

Supp Figure 2

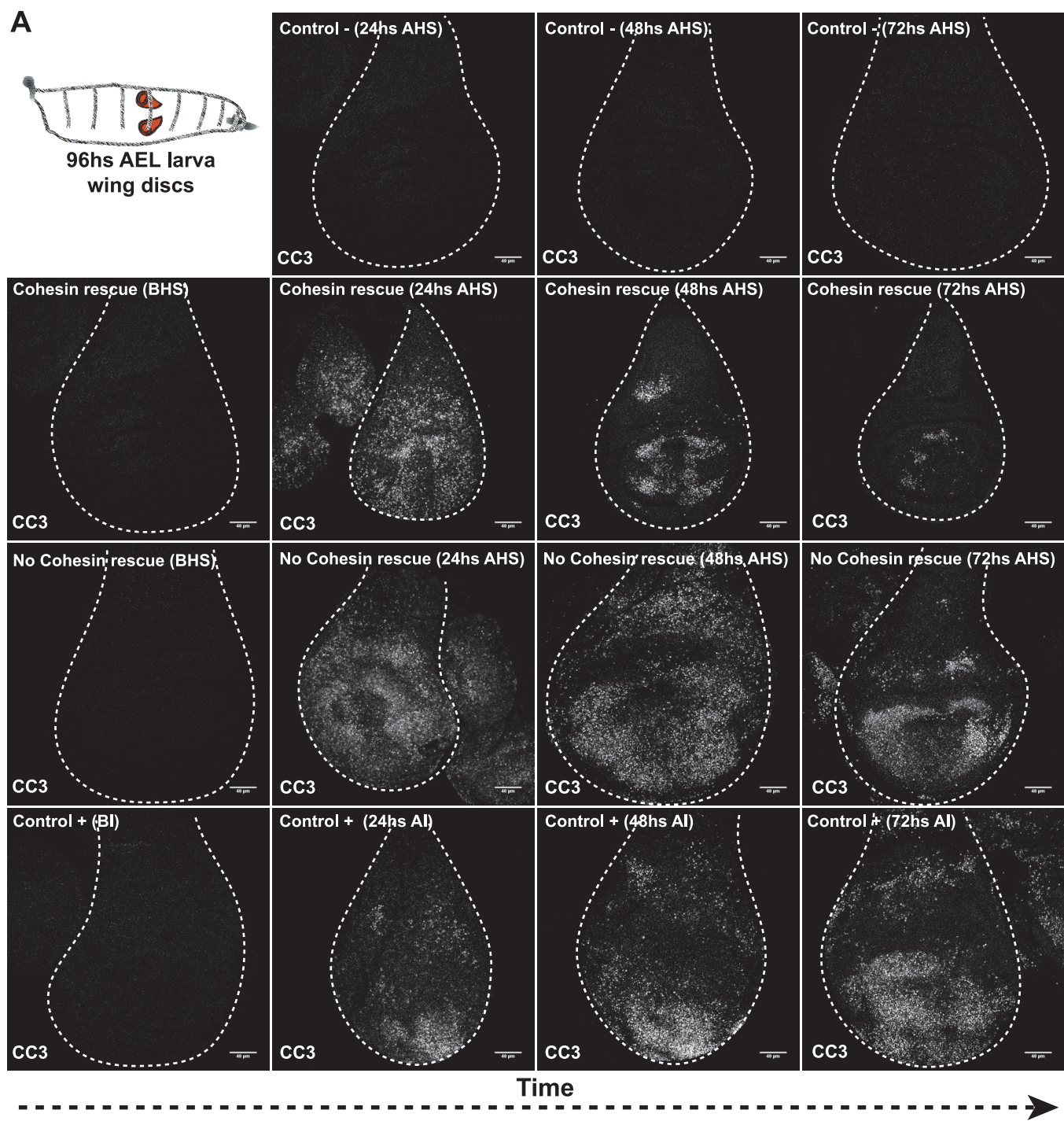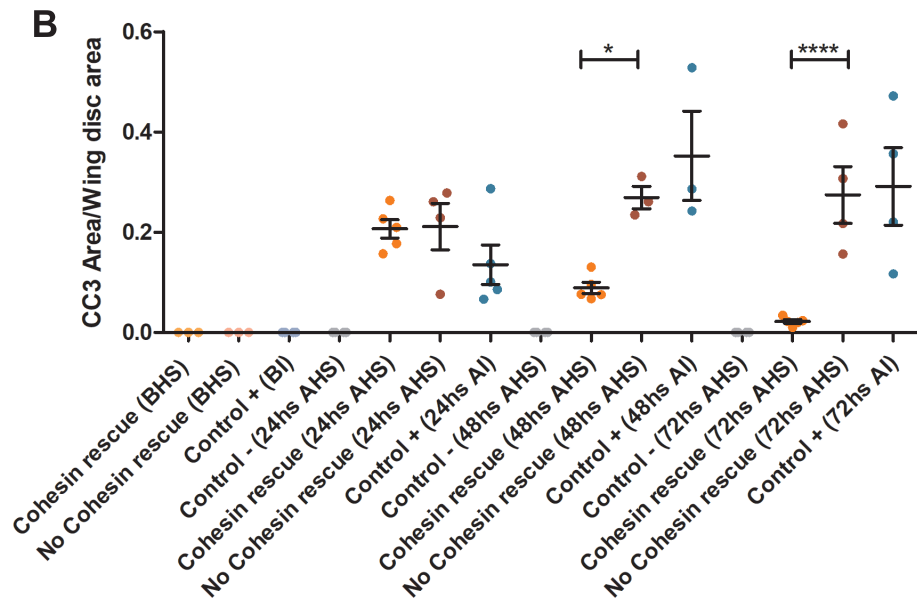

Supplement: S2 Fig — (A–B) (A) Reversible cohesin cleavage results in apoptosis in the third-instar wing discs (dashed shapes depict the wing disc areas). The amount of apoptosis per disc was measured by area of CC3 immunofluorescence at 24, 48, and 72 hours AHS. (B) Rescue of cohesin function significantly reduced the amount of apoptosis within 48 hours AHS. In contrast, chronic inactivation of cohesin complex (no cohesin rescue) displayed high levels of apoptosis through time. Control− (Control HS); Control+ (Irradiation: 4,000 rads). *P < 0.05; ****P < 0.0001. Scale bar = 40 μm. Individual numerical values for the presented graphs can be found in S2 Data. AHS, after heat-shock induction; AI, After Irradiation; BHS, Before Heat-Shock; BI, Before Irradiation; CC3, Cleaved Caspase 3; HS, heat shock; z-proj, z projection. (PDF) [file pbio.3000016.s002.pdf]

# Supp Figure 3

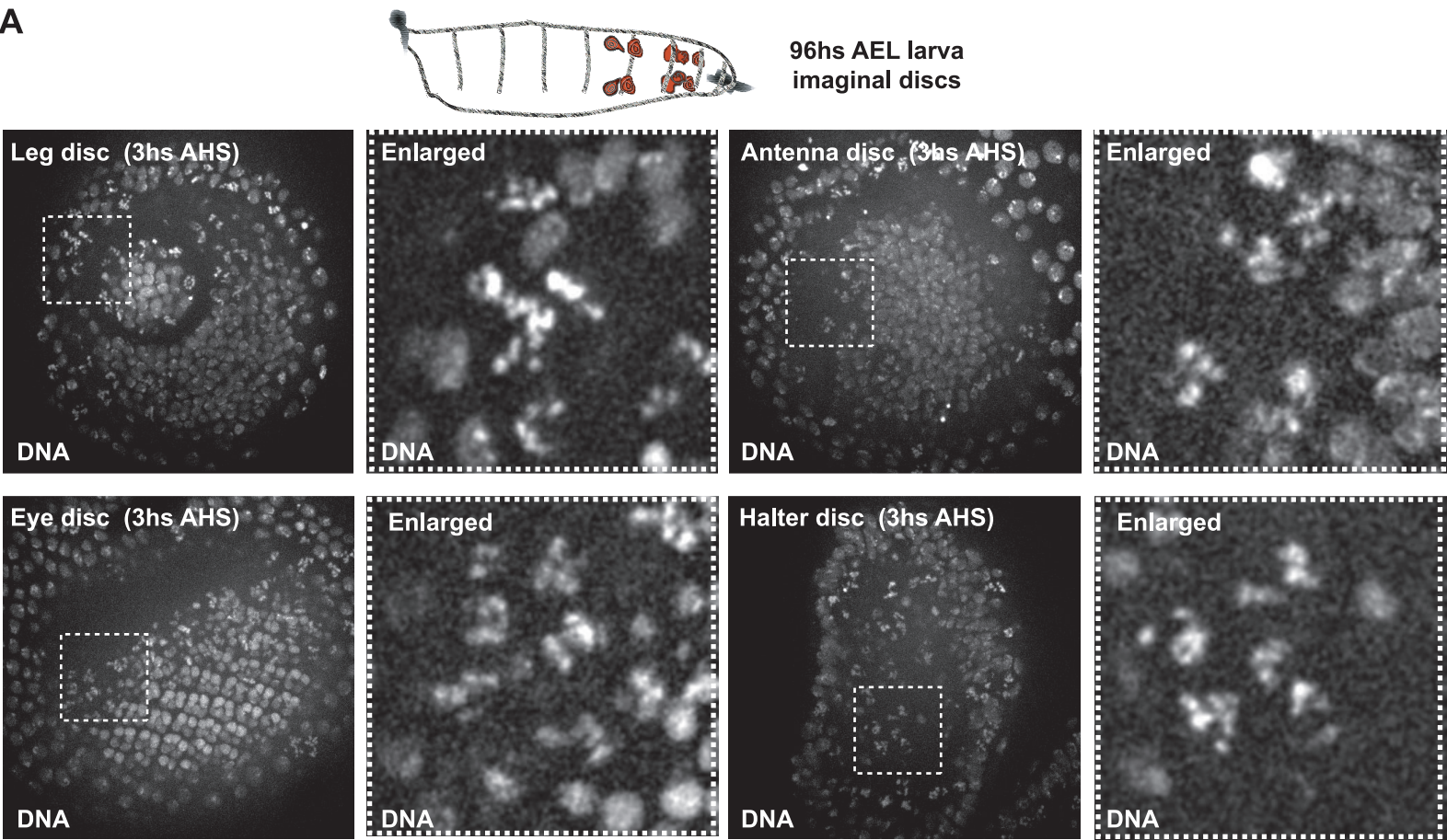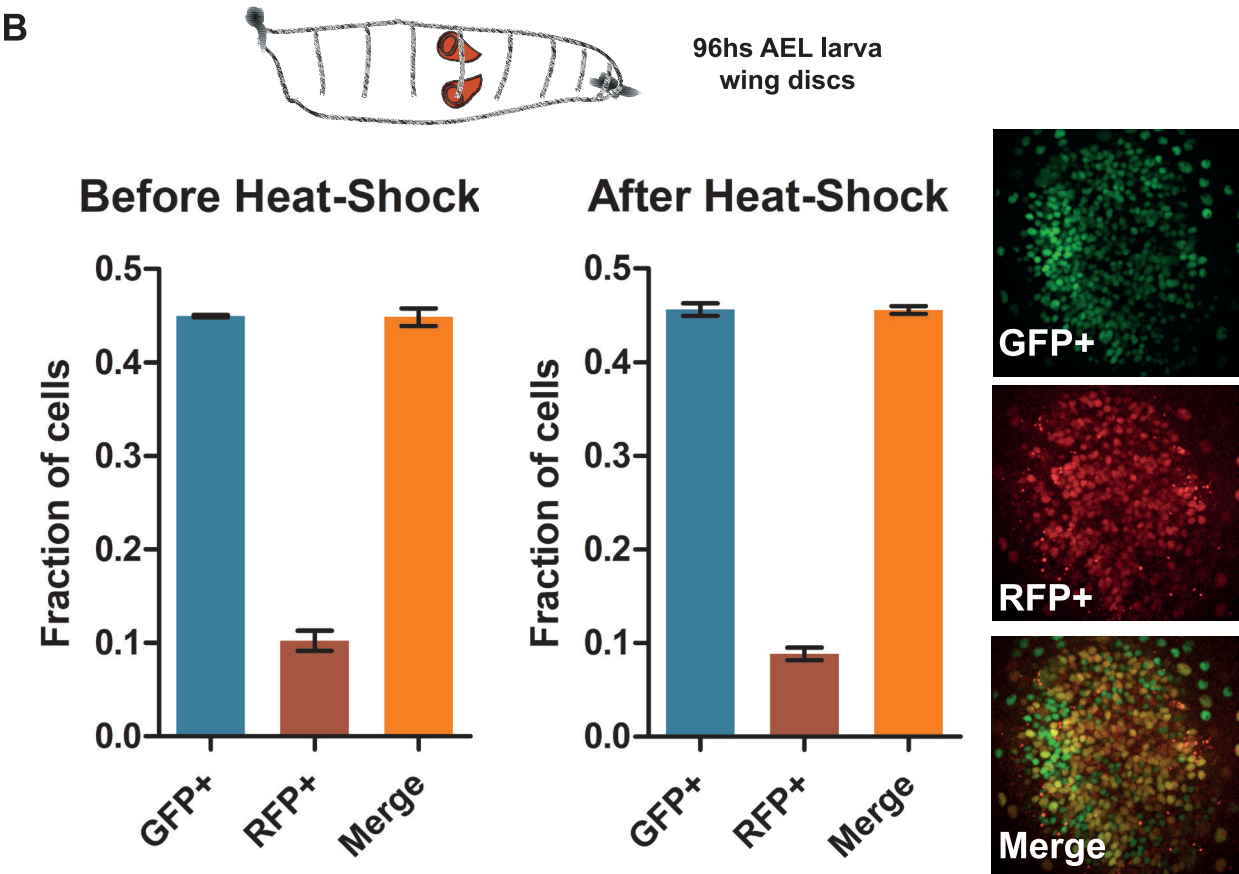

Supplement: S3 Fig — (A) Stills from live imaging of leg, eye, antennae, and haltere third-instar imaginal discs after induction of RAD21 cleavage. Dashed squares display epithelial cells from the imaginal discs undergoing mitosis with loss of cohesin (see enlarged picture). (B) The cell-cycle profile evaluation of the third-instar control wing disc with or without the heat shock, using the fly FUCCI system. The high incidence of cells affected by reversible cohesin cleavage is consistent with a high frequency of cells in G2/M in this tissue (see Merge). GFP: G1 cells; RFP: S-phase cells; Merge: G2/M Cells (n > 500, at least three wing discs analyzed). Individual numerical values for the presented graphs can be found in S2 Data. FUCCI, Fluorescence Ubiquitination Cell Cycle Indicator; GFP, green fluorescent protein; G2, Gap 2 phase; M, Mitosis; RAD21, Double-strand-break repair protein rad21 homolog; RFP, red fluorescent protein; S, Synthesis phase. (PDF) [file pbio.3000016.s003.pdf]

# Supp Figure 4

A

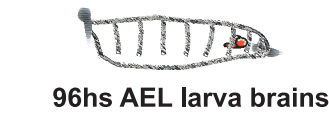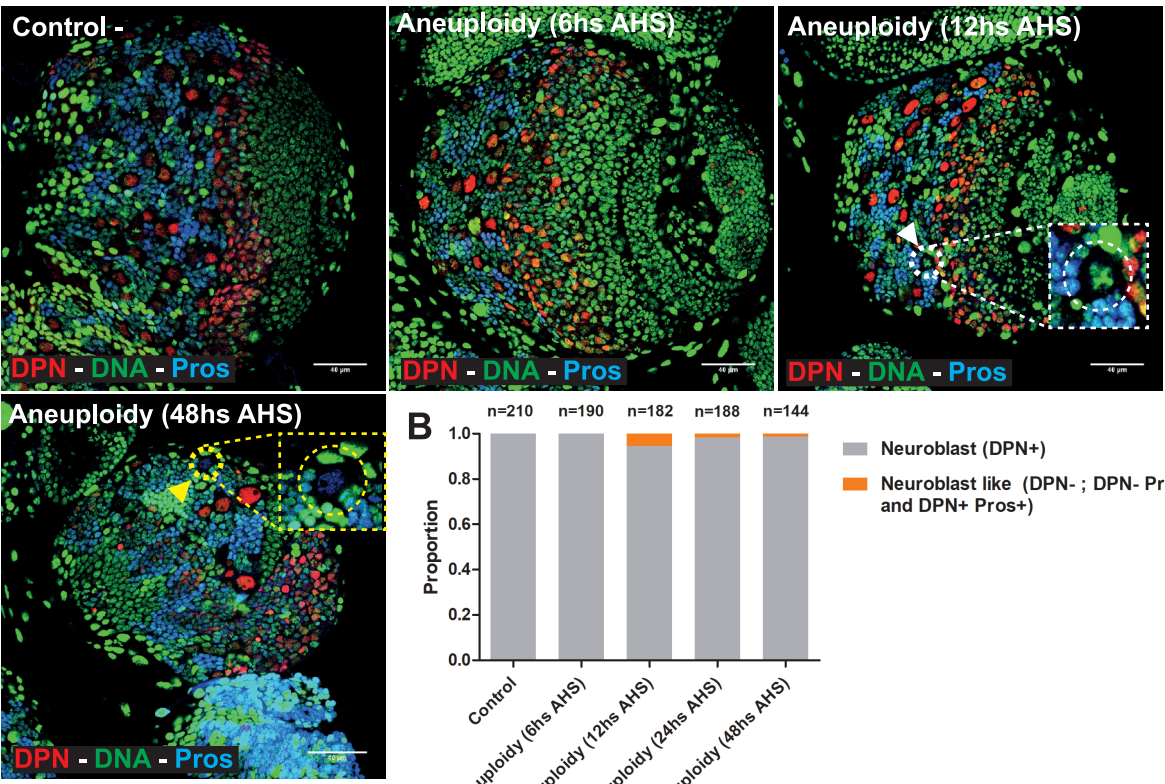

C

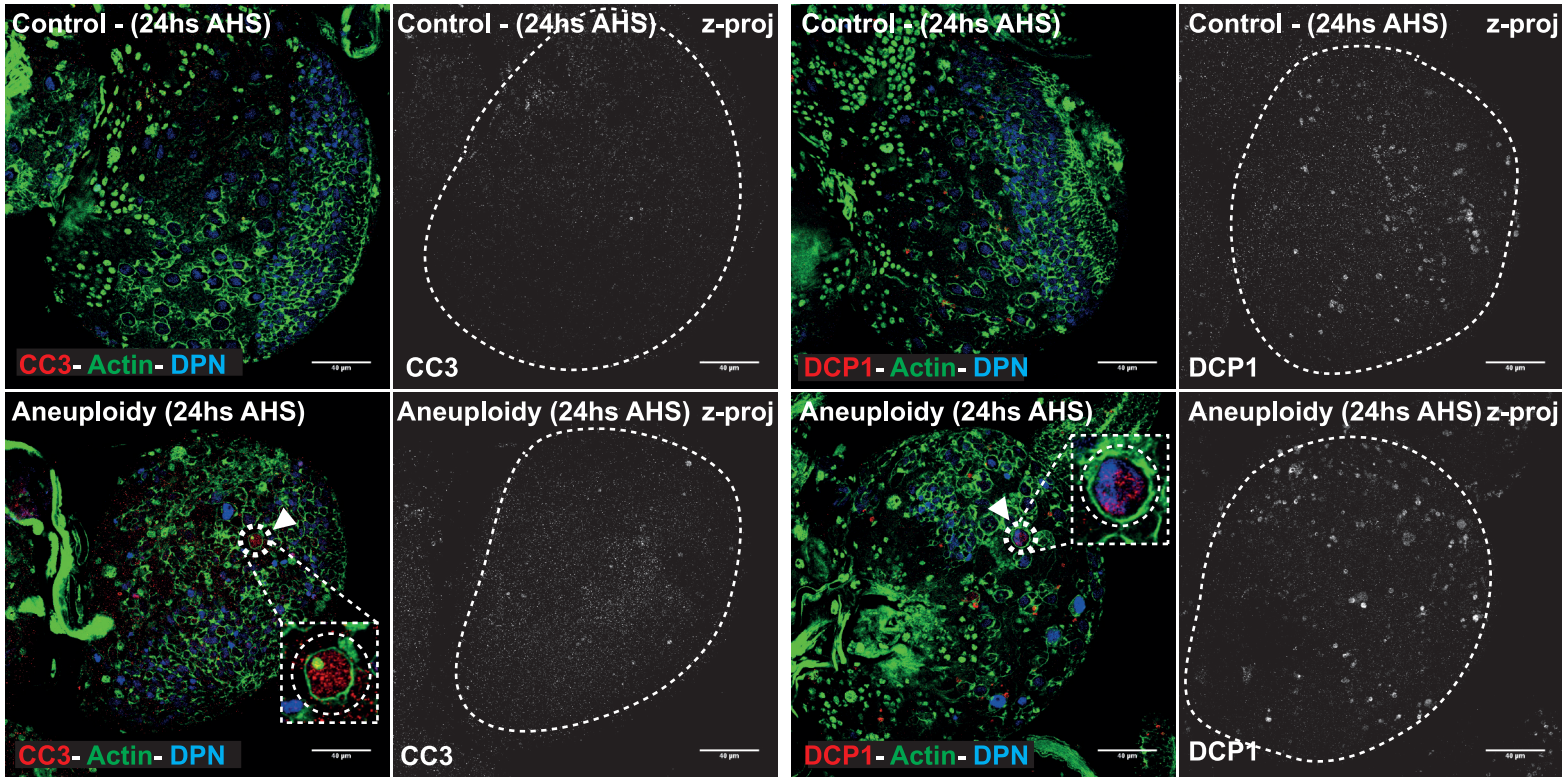

D

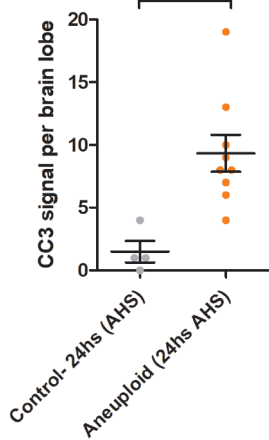

E

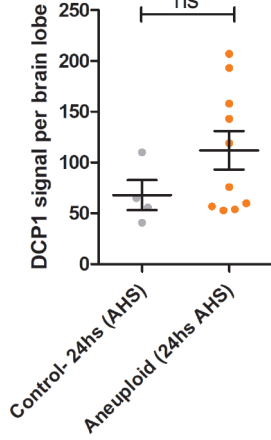

F

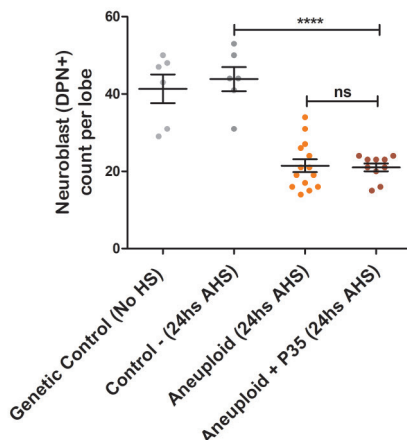

Supplement: S4 Fig — (A–B) (A) Pictures from fixed samples of third-instar larvae lobe brains stained with DPN, Pros, and Histone RFP (DNA). Induction of aneuploidy results in the loss of stem-cell identity measured by the absence of DPN (stem-cell marker, white arrowhead with dashed circle), appearance of Pros (differentiation marker, yellow arrowhead with dashed circle), or both markers together in cell nucleus with “Nbs-like shape.” (B) Percentage of loss of stem-cell identity in the neural stem-cell pool at different time points after the induction of aneuploidy. These events are observed at very low frequency. n = number of Nb-like cells. Scale bar = 40 μm. (C–E) (C) Pictures from fixed samples of third-instar larvae lobe brains stained with DPN, CC3 (death marker), DCP1 (death marker), and rhodamine phalloidin (Actin). Induction of aneuploidy results in cell death measured by the presence of CC3 or DCP1 signals (white arrowheads with dashed circles) in cells with “Nbs-like shape.” (D and E) Quantification of cell death signals CC3 and DCP1 per larvae brain lobes at 24 hours AHS. The presence of positive signal for the cell death markers in Nb-like cells is very low. **P < 0.01. Scale bar = 40 μm. (F) Quantification of Nbs at the CB in third-instar lobe brains assessed by immunofluorescence with the Nb marker DPN. Inhibition of apoptosis by overexpression of baculovirus P35 does not rescue Nb number after 24-hours–induced aneuploidy. n = number of lobe brains. ****P < 0.0001. Individual numerical values for the presented graphs can be found in S2 Data. AHS, after heat-shock induction; CB, central brain; CC3, Cleaved Caspase 3; DCP1, Death Caspase-1; DPN, Deadpan; Nb, Neuroblast ns, not significant; Pros, Prospero; RFP, red fluorescent protein. (PDF) [file pbio.3000016.s004.pdf]

# Supp Figure 5

A

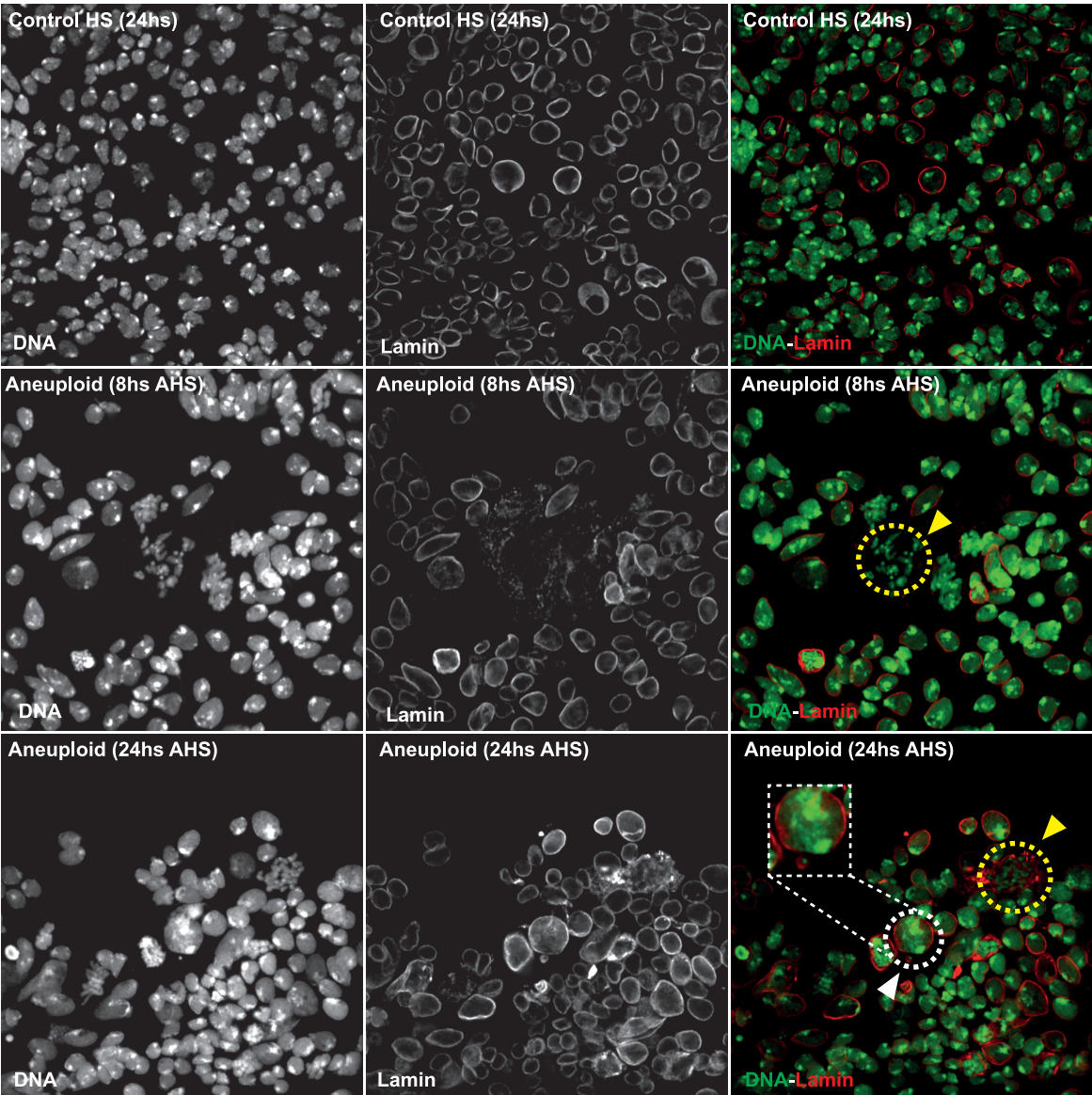

B

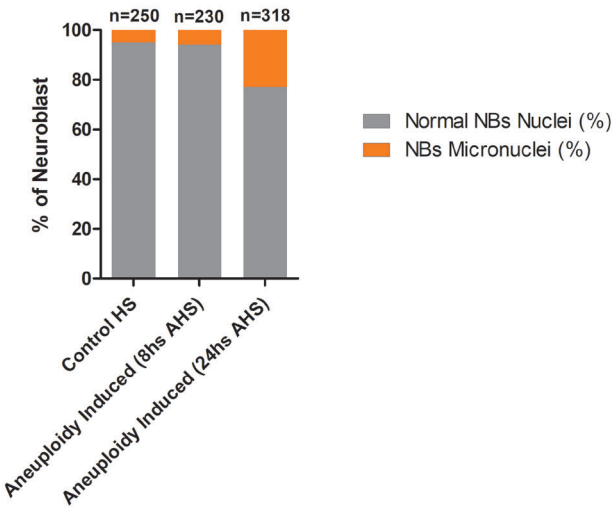

Supplement: S5 Fig — (A–B) (A) Micronuclei assessment upon aneuploidy induction. Only after 24 hours AHS was the percentage of micronuclei per Nb counts different from the control (Control HS: 5% versus aneuploidy-induced 24 hours AHS: 24%). (B) Quantification of micronuclei at the different conditions. Micronuclei were assessed by counting DNA signal (green) together with Lamin immunofluorescence (red) in spreads from brain tissues at 8 and 24 hours AHS. Micronuclei were defined as a DNA particle with enclosed-by-LAMIN staining with a perimeter (Fiji measurement) smaller than 60. Number of brains analyzed (Control HS (8 + 24 hours AHS) = 10; 8 hours AHS = 8; 24 hours AHS = 8). n = number of cells. Micronuclei are indicated by white dashed circles with arrowhead. Aneuploid Nbs are indicated by yellow circles with arrowhead. High magnification of micronuclei is shown by dashed squares. Individual numerical values for the presented graphs can be found in S2 Data. AHS, after heat-shock induction; HS, heat shock; Nb, Neuroblast. (PDF) [file pbio.3000016.s005.pdf]

## Supp Figure 6

**A**

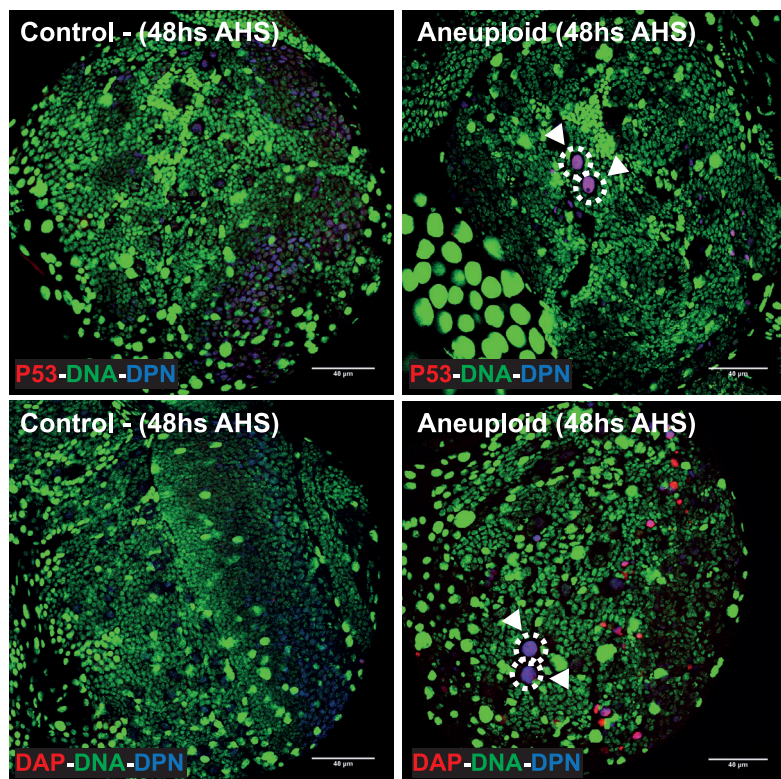

**B**

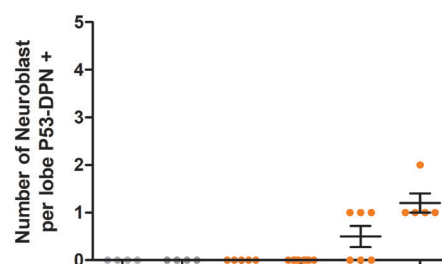

**C**

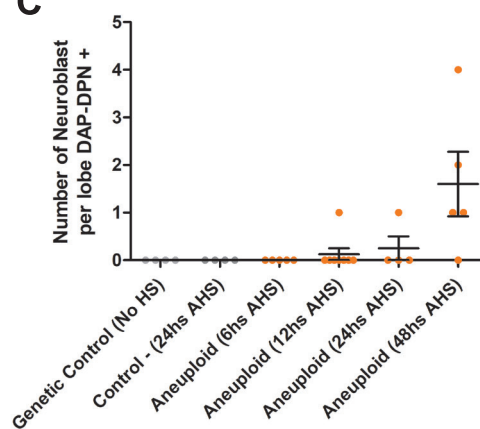

Supplement: S6 Fig — (A–C) Pictures from fixed samples of third-instar larvae lobe brains showing the immunofluorescence of canonical stress-response markers P53 and DAP together with the Nb marker DPN at 48 hours AHS. Nbs display a delayed aneuploidy stress response at 48 hours AHS (arrowheads with dashed circles). (B and C) Quantification of the kinetics of the aneuploidy-induced stress response at 6, 12, 24, and 48 hours AHS in Nbs (DPN+). Scale bar = 40 μm. Individual numerical values for the presented graphs can be found in S2 Data. AHS, after heat-shock induction; DAP, Dacapo; DPN, Deadpan; Nb, Neuroblast. (PDF) [file pbio.3000016.s006.pdf]

Supp Figure 7

A

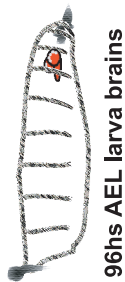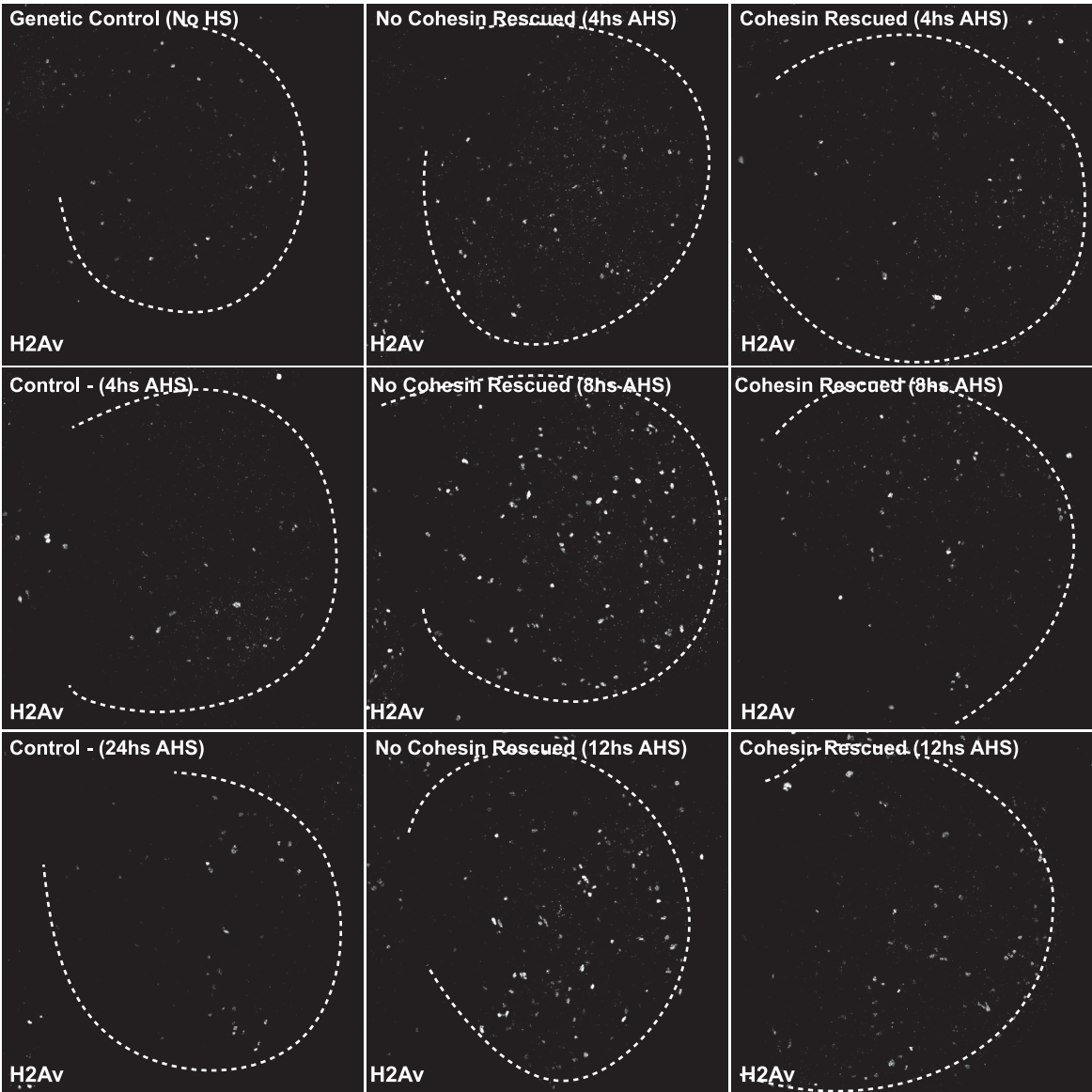

B

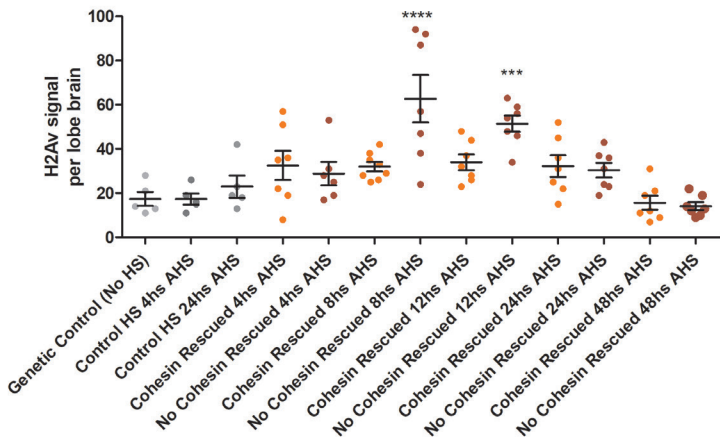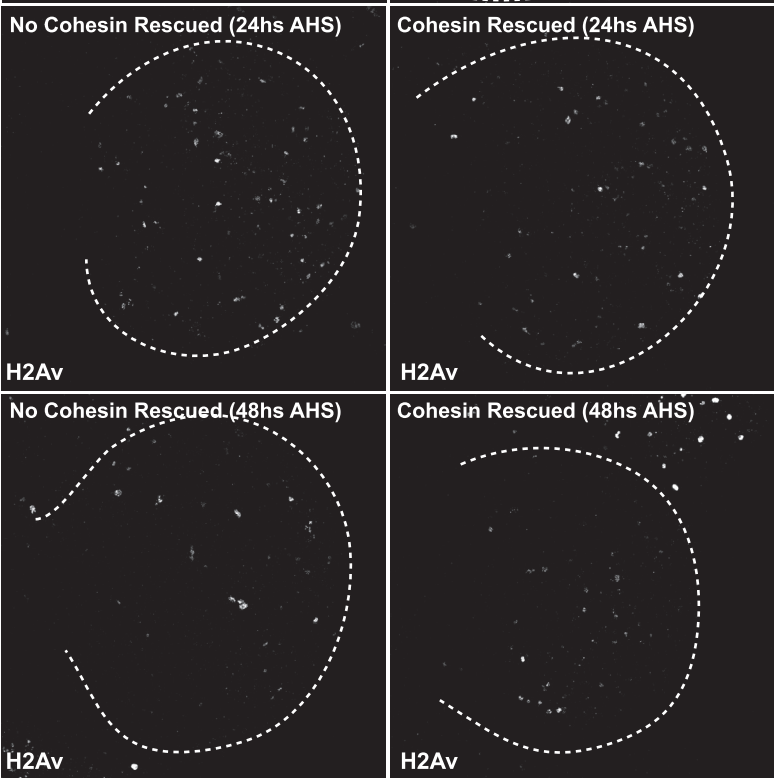

hours after induced loss of cohesin and consequent aneuploidy

Supplement: S7 Fig — (A–B) (A) Pictures from fixed samples of third-instar larvae lobe brains showing the immunofluorescence stainings with the H2Av antibody (DSBs marker in Drosophila) at 4, 8, 12, 24, and 48 hours with and without rescuing RAD21 depletion. (B) Quantification of the H2Av signal kinetics after loss of cohesin and consequent aneuploidy at 4, 8, 12, 24, and 48 hours AHS. ***P < 0.001; ****P < 0.0001 compared with no-HS control. Individual numerical values for the presented graphs can be found in S2 Data. AHS, after heat-shock induction; DSB, double strand break; HS, heat shock; H2Av, Histone H2A variant; RAD21, Double-strand-break repair protein rad21 homolog. (PDF) [file pbio.3000016.s007.pdf]

# Supp Figure 8

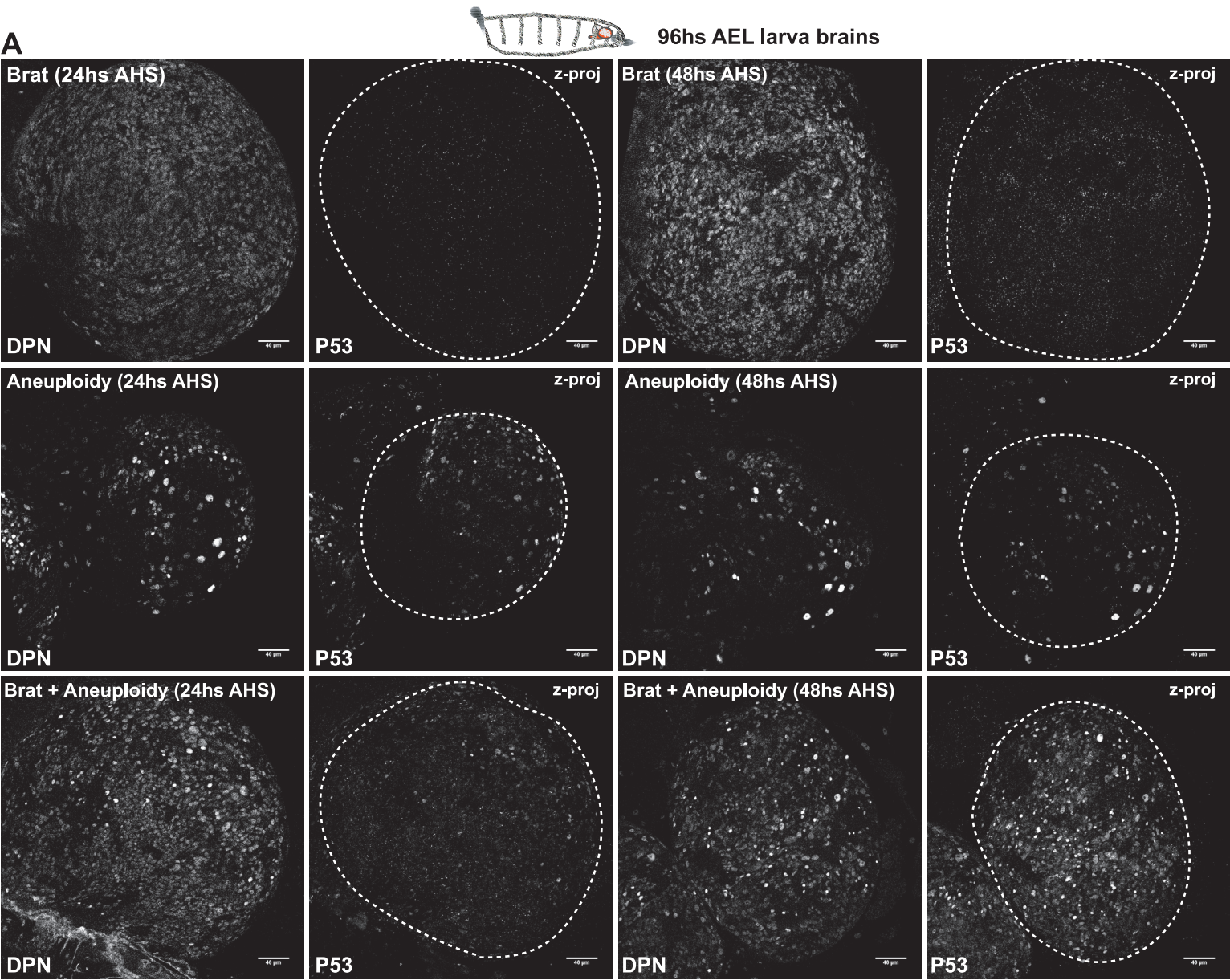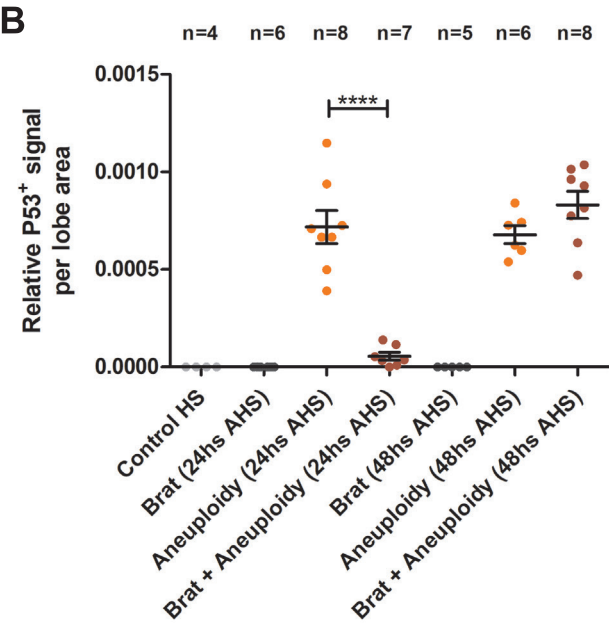

Supplement: S8 Fig — (A–B) (A) Brat mutant lobe brains showed a clear reduction in the presence of the aneuploidy-induced stress marker P53 at 24 hours AHS. (B) Quantification of relative P53-positive signal per lobe area from 24 to 48 hours AHS. n = number of lobe brains. ****P < 0.0001. Scale bar = 40 μm. Individual numerical values for the presented graphs can be found in S2 Data. AHS, after heat-shock induction; Brat, Brain tumor; z-proj, z projection. (PDF) [file pbio.3000016.s008.pdf]

Figure Supp 9

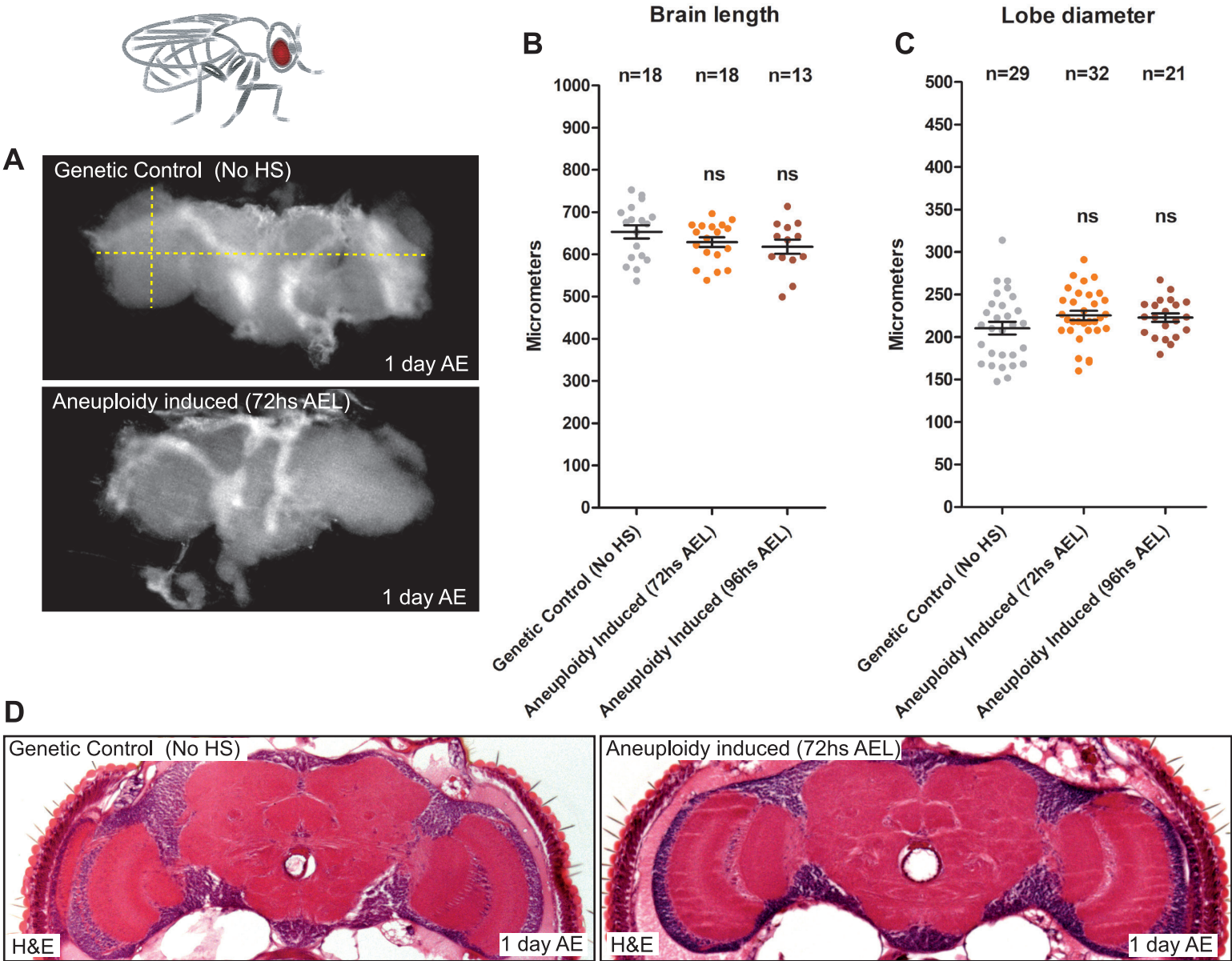

Supplement: S9 Fig — (A–C) (A) Dissected brains of adult flies from control and developmental aneuploidy-induced (72-hours–AEL heat shock) organisms. (B and C) Quantifications of lobe diameter and brain length in control and developmental aneuploidy-induced (72- and 96-hours–AEL heat shock) adult flies showed no significant differences. n = number of brains. (D) Histology analysis of brains from control and aneuploidy-induced-during–development (72-hours–AEL heat shock) adult flies, 1 day after eclosion. Frontal sections at approximately midbrain showed no signal of neurodegenerative process (vacuolization). Individual numerical values for the presented graphs can be found in S2 Data. AEL, after egg laying; HE, hematoxylin–eosin; ns, not significant. (PDF) [file pbio.3000016.s009.pdf]
